# Asymmetric synthesis of a stereopentade fragment toward latrunculins

**DOI:** 10.3762/bjoc.19.32

**Published:** 2023-04-03

**Authors:** Benjamin Joyeux, Antoine Gamet, Nicolas Casaretto, Bastien Nay

**Affiliations:** 1 Laboratoire de Synthèse Organique, Ecole Polytechnique, CNRS, ENSTA, Institut Polytechnique de Paris, 91128 Palaiseau, France; 2 Laboratoire de Chimie Moléculaire, Ecole Polytechnique, CNRS, Institut Polytechnique de Paris, 91128 Palaiseau, France

**Keywords:** allylation, aldol reaction, latrunculins, stereocontrol, total synthesis

## Abstract

Latrunculins are marine toxins used in cell biology to block actin polymerization. The development of new synthetic strategies and methods for their synthesis is thus important in order to improve, modulate or control this biological value. The total syntheses found in the literature all target similar disconnections, especially an aldol strategy involving a recurrent 4-acetyl-1,3-thiazolidin-2-one ketone partner. Herein, we describe an alternative disconnection and subsequent stereoselective transformations to construct a stereopentade amenable to latrunculin and analogue synthesis, starting from (+)-β-citronellene. Key stereoselective transformations involve an asymmetric Krische allylation, an aldol reaction under 1,5-*anti* stereocontrol, and a Tishchenko–Evans reduction accompanied by a peculiar ester transposition, allowing to install key stereogenic centers of the natural products.

## Introduction

**Figure 1:**
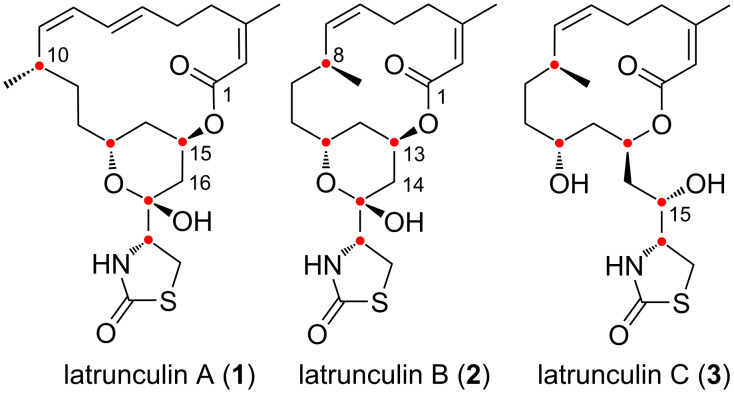
Structure of latrunculins (the red dots show the natural product stereopentade).

Latrunculins constitute a class of marine polyketide natural products isolated from Sponges like *Negombata* (= *Latrunculia) magnifica* [1–2]. They are characterized by the presence of an unsaturated fourteen- or sixteen-membered macrolactone decorated by an ʟ-cysteine-derived 2-oxo-1,3-thiazolidin-4-yl substitutent, and the presence of five stereogenic centers forming a 1,2,4,6,9-stereopentade (Figure 1). In latrunculins A (**1**) and B (**2**) three of them are embedded in a lactol ring, while latrunculin C (**3**) lacks this ring due to the reduction of C-15. The biological activities of latrunculins A and B have early been reported [3]. These compounds induce important but reversible morphological changes on mouse neuroblastoma and fibroblast cells at low concentrations such as 50 ng/ mL [2]. It was rapidly demonstrated that the toxins target the cytoskeleton and inhibit the actin polymerization by specifically sequestering the G-actin monomers with a high affinity [4], unlike cytochalasin D that targets the actin filament [5]. Structure–activity relationships have also been demonstrated thanks to the synthesis of analogues, which hardly superseded the natural product properties, highlighting the importance of the macrocycle and of the lactol ring for this biological activity (**3** is inactive) [6–7].

**Figure 2:**
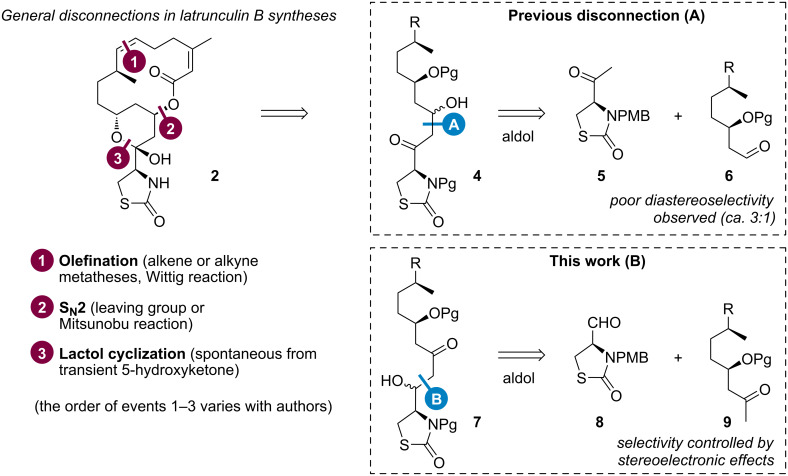
General strategy for latrunculin cycle disconnections (left), previous works towards linear precursor **4** (A), and our alternative disconnection of **7** through the aldol reaction (B).

Considering the structural features of these toxins and their valuable biological properties (**1** and **2** are nowadays commercially available as tools for cell biology), latrunculins have been appealing targets for synthetic studies. Several total syntheses of latrunculins were reported by Smith III [8–10], White [11], Fürstner [6,12] and Watson [13]. These syntheses involved similar disconnection strategies for the macrocycle or the lactol formation (Figure 2, left), and for the aldol reaction leading to **4**, using a 4-acetyl-1,3-thiazolidin-2-one **5** as ketone partner (Figure 2, route A). Strikingly, this last disconnection was adopted in all previous syntheses to form the (15,16)- or the (13,14)-bond of **1** and **2**, respectively. Conversely, we envisaged an alternative disconnection to form the (16,17)- or the (14,15)-bond of **1** and **2**, through an aldol reaction of aldehyde **8** readily available from ʟ-cysteine, leading to aldol adduct **7** (Figure 2, route B). The methyl ketone partner **9** could be formed by the oxidation of an allyl moiety introduced by the asymmetric allylation of an aldehyde derived from (+)-β-citronellene. At this stage, we can speculate that the stereocontrol of this reaction could either follow a polar Felkin–Anh model [14–16] based on chiral aldehyde partner **8** [17], or a 1,5-*anti* induction of the aldol reaction [18–20] based on chiral alkoxy partner **9**. Furthermore, it could be envisaged to reduce the resulting β-hydroxyketone **7** in a diastereoselective manner to obtain a 1,3-diol.

This synthetic strategy could thus bring new stereochemical opportunities to synthesize latrunculin analogues for chemical biology studies. In particular, our initial goal was to protect an inactive lactol-opened form of latrunculins, which could cyclize in vivo upon deprotection under a specific stimulus (light or enzyme, for instance) for biological applications. This challenge precluded the installation of the pyran ring – and the use of its well-known isomerization to set up important stereocenters [6,9] –, thus imposing the anticipated construction of key asymmetric centers. The following discussion will deal with the stereoselective synthesis of a stereopentade amenable to such latrunculin synthesis and the encountered difficulties thereof.

## Results and Discussion

**Scheme 1:**
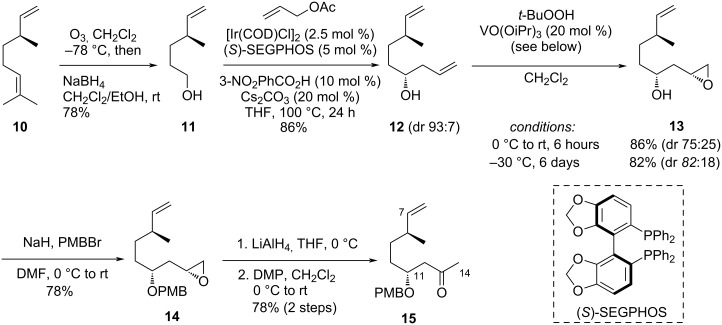
Synthesis of fragment **15** from (+)-β-citronellene (**10**).

Our synthesis started from commercially available (+)-β-citronellene (**10**). The ozonolysis of the trisubstituted double bond followed by a reductive treatment with NaBH_4_ chemoselectively afforded primary alcohol **11** in 78% yield (Scheme 1). Due to easier purification, this alcohol was preferred to the aldehyde in our synthetic route, allowing a key stereoselective Krische allylation [21–22] to be envisaged. Applying reported conditions for this allylation – in presence of allyl acetate (10 equiv), [Ir(COD)Cl]_2_ (2.5 mol %), (*S*)-SEGPHOS (5 mol %), 3-nitrobenzoic acid (10 mol %), Cs_2_CO_3_ (20 mol %) in THF at 100 °C for 24 hours – we obtained homoallylic alcohol **12** in a good 86% yield, with a diastereomeric ratio (dr) of 93:7 deduced from the NMR analysis of the methyl substituent signals in CD_3_OD (NMR spectra compared to those of a 50:50 mixture of diastereoisomers, obtained from the addition of allylmagnesium bromide onto the corresponding aldehyde). The stereochemistry of the resulting secondary alcohol was expected to be (*R*) according to Krische's studies involving (*S*)- SEGPHOS [22]. This result was secured by the NMR analysis of Mosher's esters made from (*R*)-(+)- and (*S*)-(−)-α-methoxy-α-trifluoromethylphenylacetic acid (MTPA) (see Supporting Information File 1) [23–25], confirming the installation of the C-11 stereocenter of latrunculins.

The next steps consisted in the functionalization of **12**, in view of its coupling to **8**. We first relied the chemoselective epoxidation of the homoallylic alcohol, done in presence of VO(OiPr)_3_ (20 mol %) and *t*-BuOOH to afford epoxide **13**, in 86% yield and a dr of 75:25 (measured by NMR, presumably resulting from the major diastereoisomer of **13**; minor isomers were not identified), when the reaction was performed at room temperature during 6 hours. This vanadium catalyst superseded VO(acac)_2_ in terms of yields [26–27]. Additional epoxidation attempts allowed to improve the dr to 82:18 (82% yield) when the reaction was left at −30 °C for 6 days. Unfortunately, it was not possible to set up an appropriate nucleophile through the umpolung of aldehyde **8** to react with this epoxide, which led us to envisage the following aldol strategy through ketone **15**. Attempts of Wacker reactions to produce **15** were unsuccessful on **12**, presumably due to a competition between the two olefinic parts. After protection of the secondary alcohol as a *para*-methoxybenzyl (PMB) ether (78% yield of **14**), the ketone (**15**) was installed in two steps from the epoxide (direct rearrangement attempts of the epoxide to form the ketone were unsuccessful). Thus, the epoxide was first reduced on its primary carbon in presence of LiAlH_4_, and the resulting secondary alcohol was oxidized in presence of Dess–Martin periodinane (DMP), giving ketone **15** in 78% yield over the two steps. This six-step sequence to **15** was performed in a 35% overall yield from starting material **10**.

**Scheme 2:**
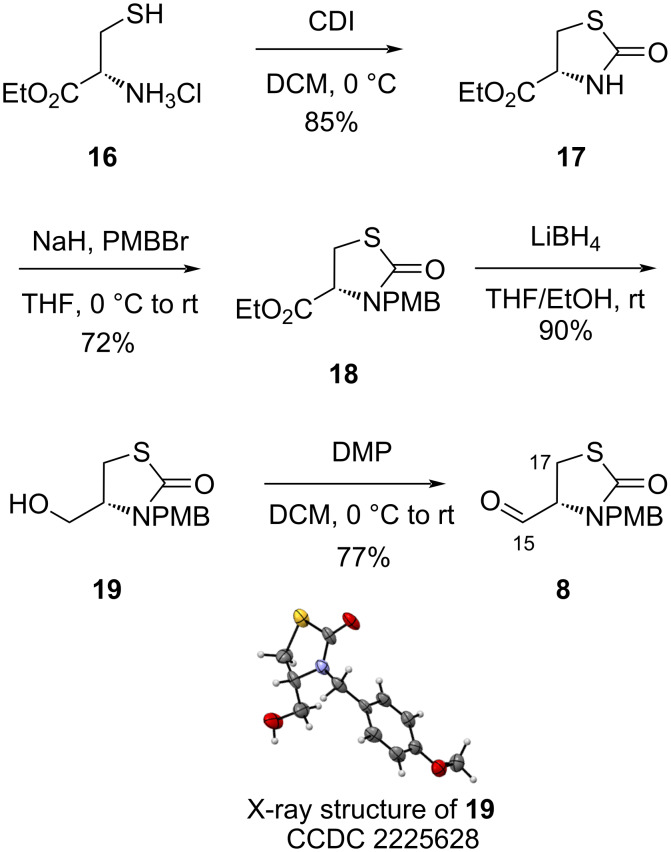
Synthesis of fragment **8** from ʟ-cysteine ethyl ester hydrochloride (**16**).

The aldehyde partner (**8**) for the aldol reaction brings the thiazolidinone heterocycle of the natural product. It was synthesized in four steps from ʟ-cysteine ester derivative **16**, first reacting with carbonyldiimidazole (CDI) to afford thiazolidinone **17** in 85% yield (Scheme 2). The nitrogen atom was protected with a PMB group in 72% yield (**18**), after deprotonation with NaH and reaction with PMBBr. The ester moiety of **18** was then chemoselectively reduced into alcohol **19** in 90% yield, in presence of LiBH_4_ to avoid the reduction of the thiazolidinone part. Finally, the aldehyde (**8**) was generated in 78% yield by oxidation in presence of DMP.

**Scheme 3:**
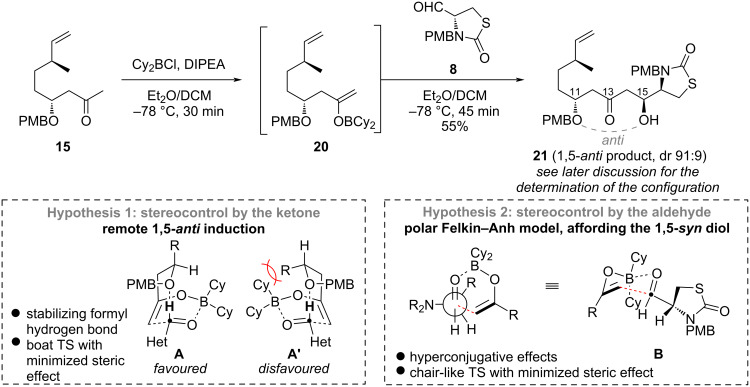
Synthesis of fragment **21** through a stereoselective aldol reaction.

The assembly of aldehyde **8** and methyl ketone **15** was envisaged through a stereoselective aldol reaction. After unsuccessful attempts of Mukaiyama aldol reactions with silyl enol ethers [28], we found that dicyclohexylboron enolate **20**, made in situ from ketone **15** and Cy_2_BCl in presence of DIPEA, performed well in the aldol reaction to furnish product **21** in 55% yield with a good dr of 91:9 (Scheme 3).

The stereocontrol of the reaction could be envisaged through two principal mechanisms. A remote stereocontrol by the nucleophile could first be expected [29], through a 1,5-*anti*-induction of the aldol stereocenter by β-alkoxy ketone **9**, leading to an (*S*)-configuration [18–20]. This control is supposed to follow a boat transition state **A** stabilized by a formyl hydrogen bond [30]. It is known to be dependent on the nature of the β-alkoxy substituent, being particularly favoured by the PMB and other aromatic groups, while being disfavoured by silyl protecting groups. Alternatively, an (*R*)-configuration of C-15 could result from a polar Felkin–Anh model controlled by aldehyde **8** through chair-transition state **B** [14–16]. To determine the configuration of C-15, we initially relied on the comparative NMR analysis of Mosher's esters [23]. Despite clear ^1^H NMR spectra, irregular values of Δδ^S–R^ precluded the unambiguous determination of the C-15 stereocenter [24]. These difficulties were attributed to the hindered character of this secondary alcohol, substituted by the thiazolidinone ring, possibly leading to a strong conformational distortion of Mosher's model. The question of the resulting stereoselectivity was thus left open for later resolution.

**Scheme 4:**
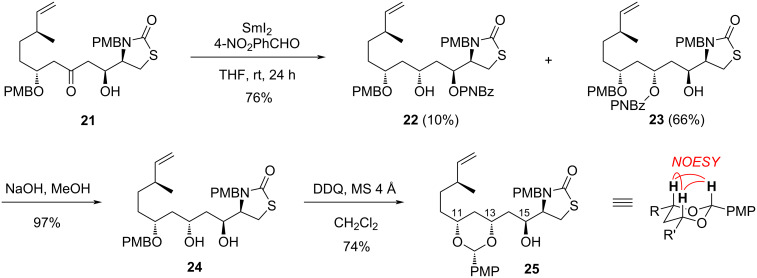
1,3-*Anti*-diastereoselective reduction of **21** with PNBz transposition, and final determination of the relative stereochemistry by NOESY experiment on **25**.

To complete this study, the 1,3-*anti*-diastereoselective reduction of β-hydroxyketone **21** was undertaken through the Evans–Tishchenko method [31–32], in presence of SmI_2_ and an aldehyde (Scheme 4). *para*-Nitrobenzaldehyde was used [33] to introduce a labile *para*-nitrobenzoate on the product, planning an easy deprotection of the alcohol. This would also pave the way to an orthogonal manipulation of protecting groups on the stereopentade, in view of designing molecular tools for biological purpose. The reduction took place in 76% yield with complete stereoselectivity. However, a mixture of two inseparable products was obtained, containing the expected but minor alcohol **22** (10%), and more surprisingly the major isomer **23** (66%). This compound results from the transposition of the *para*-nitrobenzoyl (PNBz) group onto the 13-OH, which could be favoured by the steric hindrance of C-15 and a possible π–π stacking with the OPMB group.

These PNBz esters were readily hydrolyzed to furnished diol **24** in 97% yield. The oxydation of the PMB group, in presence of DDQ under anhydrous conditions [18], gratifyingly afforded acetal **25** in 74% yield, whose stereochemical assignment by NOESY NMR experiment showed the *syn* stereochemistry of the acetal. By deduction, it was confirmed that the asymmetric boron aldol reaction between **8** and **15** proceeded through a 1,5-*anti* induction by the ketone to form **21**. Most importantly, compounds **22**–**25** bear the (11*R*,13*R*) configuration of latrunculins (**1** and **2**).

## Conclusion

A straightforward synthesis of a stereopentade intermediate towards latrunculins and lactol-opened analogues was achieved with high stereoselectivity. Starting from the chiral pool bringing the 8-methyl substituent, the secondary alcohol on C-11 was stereoselectively introduced by the Krische allylation of alcohol **11**. The next key step consisted in an aldol reaction of ketone **15** onto aldehyde **8**, which proceeded with a high stereocontrol resulting from a 1,5-*anti* induction by the nucleophile leading to product **21**, and excluding a Felkin–Anh control by the aldehyde. This reaction validates a unique disconnection among latrunculin synthetic strategies and avoids the construction of a 4-acetyl-1,3-thiazolidin-2-one. Finally, this β-hydroxyketone was submitted to the SmI_2_-mediated Evans–Tishchenko reduction, which was performed with full 1,3-*anti-*stereocontrol but surprisingly resulted in the ester transposition to predominantly form alcohol **23** in good yields. This last reduction allowed to install the key (11*R*,13*R*) configuration of latrunculins.

## Supporting Information

Crystallographic data of compound **19** were deposited in the Cambridge Crystallographic Data Center under the CCDC number 2225628.

File 1 Experimental procedures, compound characterizations and spectra.

File 2 Crystallographic Information File of compound **19**.
