# Supplementary material for: Asymmetric synthesis of a stereopentade fragment toward latrunculins
Source: Beilstein J Org Chem. 2023 Apr 3;19:428–33. doi: 10.3762/bjoc.19.32 (PMC10113517; doi:10.3762/bjoc.19.32)

BEILSTEIN JOURNAL OF ORGANIC CHEMISTRY

Supporting Information

for

Asymmetric synthesis of a stereopentade fragment toward latrunculins

Benjamin Joyeux, Antoine Gamet, Nicolas Casaretto and Bastien Nay

Beilstein J. Org. Chem. **2023,** *19,* 428–433. doi:10.3762/bjoc.19.32

Experimental procedures, compound characterizations and spectra

License and Terms: This is a supporting information file under the terms of the Creative Commons Attribution License (https://creativecommons.org/licenses/by/4.0). Please note that the reuse, redistribution and reproduction in particular requires that the author(s) and source are credited and that individual graphics may be subject to special legal provisions.
The license is subject to the *Beilstein Journal of Organic Chemistry* terms and conditions: (https://www.beilstein-journals.org/bjoc/terms)

Content

General information S2

Compound preparation and characterization S3

Crystallographic data for compound 19 ....S19

References S25

Copies of NMR spectra..S26

General information

All reactions were performed in flame-dried flasks fitted with rubber septa under a positive pressure of argon. DCM and Et_2O were purified using a MB-SPS 800 (MBraun Solvent Purification System). THF was distilled over sodium and benzophenone. Anhydrous DMF was purchased from Merck. Other solvents were purchased from suppliers and were of technical quality. Petroleum ether refers to the fraction of petroleum boiling between 40 and 60 °C. Reagents were purchased and used without further purification except for allyl acetate and DiPEA, which were distilled.

Analytical TLC were carried out on aluminum plates coated with Merck F254 silica gel 60 and visualized by exposure to 240 nm UV light and/or exposure to a basic solution of potassium permanganate followed by heating. Column flash chromatography was performed using Merck PLC silica gel 60.

Infrared spectra were recorded on a Perkin Elmer Spectrum Two FTIR equipped with a Jasco ATR. Absorption maxima ($\tilde{\nu}$) are reported in wavenumbers (cm^{-1}). High resolution mass spectra (HRMS) were obtained on a Brucker tims-TOF mass spectrometer (ESI+) and reported as *m*/*z*. Optical rotary powers were recorded on an Anton Paar MCP 100 polarimeter, sample concentrations (*c*) are given in g/100 mL. Melting points were measured by a Stuart SMP40 automatic melting point apparatus.

Nuclear magnetic resonance (NMR) spectra (^1H, ^{13}C, DEPT-135, COSY, NOESY, HSQC, HMBC) were recorded at 25 °C with a Brucker Avance 400 spectrometer (^1H at 400 MHz and ^{13}C at 100 MHz). For $CDCl_3$ solutions, chemical shifts are reported as parts per million (ppm) referenced to the residual proton or carbon peak of the solvent ($CDCl_3$: δ_H = 7.26 and δ_C = 77.1). Coupling constants are reported in Hertz (Hz). Data for ^1H spectra are reported as follows: chemical shift ppm, referenced to protium (br s: broad singlet or s: singlet or d = doublet or t: triplet or q: quadruplet or m: multiplet, coupling constants J in Hz, integration, and attribution). All carbon spectra were recorded as ^1H-decoupled or DEPT-135 (Distortionless Enhancement by Polarization Transfer at 135°).

Compound preparation and characterization

(*S*)-4-Methylhex-5-en-1-ol (11)

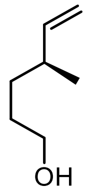

11

A three-necked round bottom flask was charged with dry CH_2Cl_2 (60 mL) and (+)-β-citronellene (**10**, 1.3 mL, 7.14 mmol, 1 equiv). The solution was cooled down to −78 °C and a stream of ozone was bubbled for 55 min. The reaction was followed by TCL until near full consumption of the starting material. Without waiting for the full disappearence of **10** to avoid over-oxidation of the second double bond, $NaBH_4$ was added (570 mg, 21.4 mmol, 3.0 equiv) followed EtOH (5 mL). The reaction was left at room temperature for 3 h 30. The reaction was carefully quenched with a 1 M solution of HCl, the product was extracted with DCM (2 × 50 mL), washed with brine, dried over $MgSO_4$, filtered and concentrated under reduced pressure. Purification by column chromatography (pentane/Et_2O: 80/20) yielded product **11** as a transparent oil (638 mg, 78% yield). Characterization data were in accordance with those of the literature [1,2].

$[\alpha_D]^{25}$ = +13.1 (c 0.137, $CHCl_3$) [Lit.[1] +16.7, c 2.05, $CHCl_3$].

^{1}H NMR (400 MHz, $CDCl_3$) δ 5.68 (ddd, J = 17.2, 10.3, 7.6 Hz, 1H, H_5), 4.96 (ddd, J = 17.2, 1.9, 1.2 Hz, 1H, H_{6a}), 4.92 (ddd, J = 10.3, 1.9, 0.9 Hz, 1H, $1H_{6b}$), 3.63 (t, J = 6.6 Hz, 2H, H_1), 2.20 – 2.07 (m, 1H, H_4), 1.62 – 1.50 (m, 2H, H_2), 1.41 – 1.29 (m, 2H, H_3), 1.00 (d, J = 6.7 Hz, 3H, 4-CH_3).

^{13}C NMR (101 MHz, $CDCl_3$) δ 144.6 (C_5), 112.9 (C_6), 63.3 (C_1), 37.8 (C_4), 32.7 (C_3), 30.6 (C_2), 20.4 (4-CH_3).

IR (ATR): $\tilde{\nu}$ = 3320, 2077, 2932, 2867, 1639, 1454, 1418, 1373, 1288, 1203, 1056, 993, 908, 680.

(4*R*,7*S*)-7-Methylnona-1,8-dien-4-ol (12)

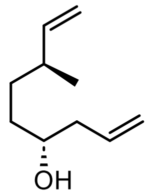

12 (*d.r.* 93:7)

A tube was charged with 29 mg of $[Ir(COD)Cl]_2$ (0.04 mmol, 2.5% equiv), 54 mg of (*S*)-SEGPHOS (0.09 mmol, 5% equiv), 29 mg of 3-nitrobenzoic acid (0.18 mmol, 10% equiv) and 114 mg of Cs_2CO_3 (0.35 mmol, 20% equiv). The argon atmosphere was restored by a cycle of vacuum and argon and a solution of 200 mg of **11** (1.75 mmol, 1 equiv) and 1.9 mL of allyl acetate (17.5 mmol, 10 equiv) in 4 mL of distilled THF was added. The tube was sealed and the reaction heated at 100 °C during 24 h. After cooling, the solution was carefully concentrated under reduced pressure and purification by flash column chromatography (pentane/Et_2O: 90/10) yielded 231 mg of product **12** as a light-yellow oil (yield: 86 %, dr: 93/7). The stereochemistry of the major alcohol was deduced from the NMR analysis of the Mosher esters **S1** and **S2** (see next paragraph).

$[\alpha_D]^{25}$= +28.1 (c 0.274, $CHCl_3$).

^{1}H NMR (400 MHz, $CDCl_3$) δ 5.82 (dddd, J = 17.6, 9.6, 7.9, 6.5 Hz, 1H, H_2), 5.67 (ddd, J = 17.2, 10.3, 7.6 Hz, 1H, H_8), 5.16 - 5.13 (m, 1H, H_{1a}), 5.13 – 5.10 (m, 1H, H_{1b}), 4.95 (ddd, J = 17.2, 1.9, 1.1 Hz, 1H, H_{9a}), 4.92 (ddd, J = 10.3, 1.9, 0.9 Hz, 1H, H_{9b}), 3.62 (m, 1H, H_4), 2.37 – 2.24 (m, 1H, H_{3a}), 2.21 – 2.05 (m, 2H, H_{3b} & H_7), 1.59 (d, J = 4.2 Hz, 1H, OH), 1.52 – 1.41 (m, 3H, H_5 & H_{6a}), 1.38 – 1.24 (m, 1H, H_{6b}), 1.00 (d, J = 6.7 Hz, 3H, 7-CH_3).

^{13}C NMR (101 MHz, $CDCl_3$) δ 144.6 (C_8), 135.0 (C_2), 118.3 (C_1), 112.9 (C_9), 70.8 (C_4), 42.1 (C_3), 37.9 (C_7), 34.5 (C_5), 32.6 (C_6), 20.4 (7-CH_3).

IR (ATR): $\tilde{\nu}$ = 3342, 3076, 2938, 2864, 1639, 1454, 1417, 1373, 1293, 993, 908, 680, 638.

HRMS (ESI+): Calculated for $C_{10}H_{19}O^+$ [MH^+]: 155.1430; Found: 155.1432.

NMR analysis of Mosher esters of alcohol 12

Mosher esters **S1** and **S2** were prepared from **12** following the following procedure:

A round bottom flask was charged with 50 mg of (*R*)- or (*S*)-α-methoxy-α-trifluoromethylphenylacetic acid (MTPA, or Mosher's acid) (0.21 mmol, 1 equiv), 10 mL

of *n*-hexane and 16 µL of DMF (0.21 mmol, 1.0 equiv). To this solution was added 85 µL of $(COCl)_2$ (1.0 mmol, 4.7 equiv) and the reaction was stirred at room temperature for 1 hour. The opaque solution was filtered and evaporated under argon flow. The crude acyl chlorides were used as such in the next step:

A round bottom flask was charged with 5 mg (0.03 mmol, 1 equiv) of **12**, 0.1 mL of dry CH_2Cl_2, 1 mg of DMAP (0.008 mmol, 20% equiv) and 10 µL of NEt_3 (0.07 mmol, 2.2 equiv). The solution was placed at 0 °C and 0.5 mL of a 0.1 M solution of the acyl chloride (0.05 mmol, 1.4 equiv) in CH_2Cl_2 was added. The reaction was kept at 0 °C for 1 hour then left at room temperature for another 2 h. The solution was concentrated under reduced pressure and the product isolated by column chromatography (pentane/Et_2O: 90/10).

(4*R*,7*S*)-7-Methylnona-1,8-dien-4-yl (*R*)-3,3,3-trifluoro-2-methoxy-2-phenyl-propanoate (S1)

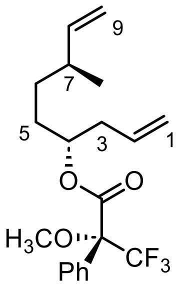

S1

^{1}H NMR (400 MHz, CDCl$_3$) δ 7.59 – 7.53 (m, 2H, PhH), 7.41 – 7.35 (m, 3H, PhH), 5.75 (ddt, J = 16.9, 10.5, 7.0 Hz, 1H, H_2), 5.52 (ddd, J = 16.9, 10.6, 7.8 Hz, 1H, H_8), 5.21 – 5.13 (m, 1H, H_4), 5.13 – 5.07 (m, 2H, H_1), 4.94 – 4.87 (m, 2H, H_9), 3.56 (q, J = 1.3 Hz, 3H, OCH_3), 2.45 – 2.33 (m, 2H, H_3), 2.07 – 1.96 (m, 1H, H_7), 1.61 – 1.53 (m, 2H, H_5), 1.21 – 1.12 (m, 2H, H_6), 0.91 (d, J = 6.7 Hz, 3H, 7-CH_3).

(4*R*,7*S*)-7-Methylnona-1,8-dien-4-yl (*S*)-3,3,3-trifluoro-2-methoxy-2-phenyl-propanoate (S2)

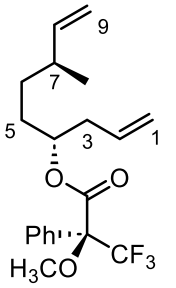

S2

^{1}H NMR (400 MHz, $CDCl_3$) δ 7.57 – 7.51 (m, 2H, PhH), 7.43 – 7.37 (m, 3H, PhH), 5.71 – 5.53 (m, 2H, H_8 & H_2), 5.17 – 5.10 (m, 1H, H_4), 5.07 – 4.98 (m, 2H, H_1), 4.95 (ddd, J = 17.2, 1.8, 1.1 Hz, 1H, H_{9a}), 4.93 (ddd, J = 10.3, 1.8, 0.9 Hz, 1H, H_{9b}), 3.54 (q, J = 1.2 Hz, 3H, OCH_3), 2.34 (ddt, J = 7.3, 6.3, 1.3 Hz, 2H, H_3), 2.15 – 2.04 (m, 1H, H_7), 1.71 – 1.57 (m, 2H, H_5), 1.38 – 1.25 (m, 2H, H_6), 0.97 (d, J = 6.7 Hz, 3H, 7-CH_3).

The two compounds were analyzed by ^{1}H NMR and the chemical shifts of the two esters were compared [3]. The protons of the C5-C9 chain on the carbinol ester were found more shielded (upfield-shifted) with the (*R*)-MTPA ester **S1** than with the (*S*)-MTPA ester **S2** (Table S1), showing an anisotropic effect of the phenyl group on the same side. Conversely, the protons of the C1–C3 substituent were found less shielded. This observation indicates an (*R*)-configuration at C4. A copy of NMR spectra is shown in Figure 1.

Table 1. NMR analysis of Mosher's esters **S1** and **S2**. The chemical shifts (ppm) for each ester and their differences [(*R*) – (*S*)] are shown. The figure shows the shielding effect in preferred Mosher's ester conformers.

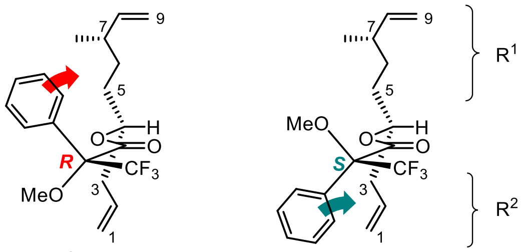

S1 **S2**

	R^1						R^2		
**proton**	**5**	**6**	**7**	**7-CH_3**	**8**	**9**	**1**	**2**	**3**
**S1 (*R* ester)**	1.57	1.16	2.02	0.91	5.52	4.90	5.11	5.75	2.41
**S2 (*S* ester)**	1.63	1.31	2.10	0.97	5.59	4.94	5.02	5.64	2.35
**$\Delta_{(R)-(S)}$**	−0.06	−0.15	−0.08	−0.06	−0.07	−0.04	+0.09	+0.09	+0.06

Figure 1: ^{1}H NMR spectra (400 MHz) of Mosher's ester **S1** (top) and **S2** (bottom) in $CDCl_3$ (calibrated on residual chloroform peak at 7.26 ppm).

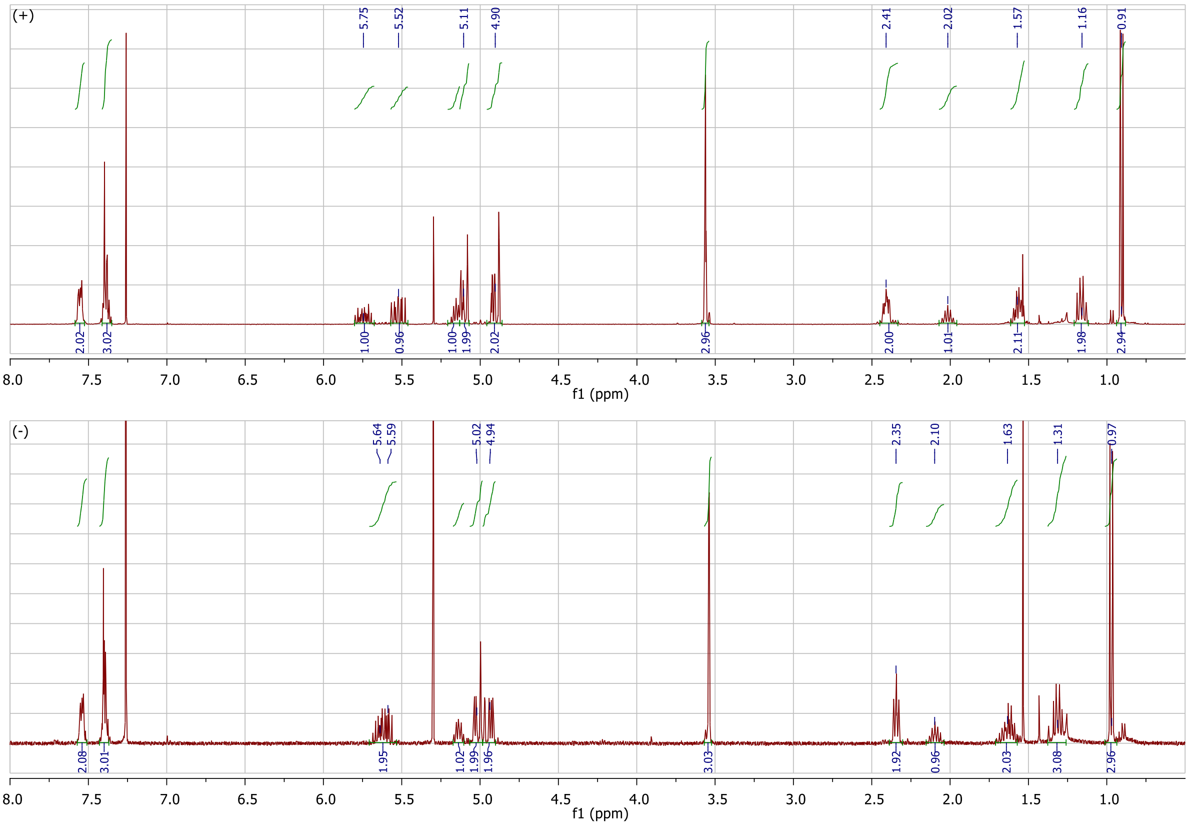

(2*R*,4*R*,7*S*)-1,2-Epoxy-7-methylnona-8-en-4-ol (13)

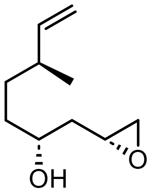

13 (*d.r.* 75:25)

A round bottom flask was charged with 1.17 g (7.60 mmol, 1 equiv) of **12**, 15 mL of dry DCM and 350 µL of $V(O)(OiPr)_3$ (1.52 mmol, 20 mol %). The solution was placed at −30 °C and 3.3 mL of a 5 M solution of di-*tert*-butylperoxide in decane (16.7 mmol, 2.2 equiv) was added. The solution was stirred at −30 °C for 6 days and quenched with a saturated solution of $Na_2S_2O_3$. The product was extracted with DCM, washed with brine, dried over $MgSO_4$, filtered and concentrated under reduced pressure. Purification by column chromatography (pentane/Et_2O: 85/15 to 60/40) yielded 1.07 g of product **13** as a transparent oil (yield: 82%, dr 82:18). The yield and reaction time could be optimized at 86% and 6 hours when the reaction was performed at room

temperature, yet giving a lower dr of 75:25. The relative stereochemistry was deduced from the known reaction mechanism and similar examples [4].

[α_D]^{25}= +53.1 (c 0.096, $CHCl_3$).

^{1}H NMR (400 MHz, $CDCl_3$) (major stereoisomer) δ 5.67 (ddd, J = 17.2, 10.3, 7.7 Hz, 1H, H_8), 4.96 (ddd, J = 17.2, 1.9, 1.2 Hz, 1H, H_{9a}), 4.92 (ddd, J = 10.3, 1.9, 0.8 Hz, 1H, H_{9b}), 3.92 – 3.83 (m, 1H, H_4), 3.08 (dtd, J = 7.5, 4.1, 2.8 Hz, 1H, H_2), 2.78 (dd, J = 4.8, 4.1 Hz, 1H, H_{1a}), 2.50 (dd, J = 4.8, 2.8 Hz, 1H, H_{1b}), 2.19 – 2.07 (m, 1H, H_7), 2.02 (d, J = 2.8 Hz, 1H, OH), 1.85 (ddd, J = 14.5, 6.7, 2.8 Hz, 1H, H_{3a}), 1.55 – 1.40 (m, 4H, H_{3b} & H_5 & H_{6a}), 1.40 – 1.31 (m, 1H, H_{6b}), 1.01 (d, J = 6.7 Hz, 3H, 7-CH_3).

^{13}C NMR (101 MHz, $CDCl_3$) (major stereoisomer) δ 144.5 (C_8), 113.1 (C_9), 70.8 (C_4), 50.8 (C_2), 46.7 (C_1), 39.9 (C_3), 37.9 (C_7), 35.2 (C_5), 32.4 (C_6), 20.5 (7-CH_3).

IR (ATR): $\tilde{\nu}$ = 3409, 2924, 2864, 2087, 1638, 1454, 1411, 1373, 1258, 1090, 995, 909, 829, 749, 682.

HRMS (ESI+): Calculated for $C_{10}H_{18}O_2Na^+$ [M + Na^+]: 193.1199; Found: 193.1193.

(2*R*,4*R*,7*S*)-1,2-Epoxy-7-methyl-4-(4-methoxybenzyloxy)nona-8-ene (14)

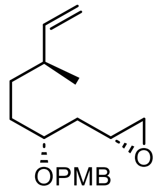

14

A round bottom flask was charged with 195 mg (1.15 mmol, 1 equiv) of **13** and 6.0 mL of dry DMF. The solution was placed at 0 °C and 46 mg of NaH (60% on mineral oil, 1.15 mmol, 1.0 equiv) was added. The solution was stirred at 0 °C for 5 minutes and 198 µL of freshly prepared PMBBr[1] (1.37 mmol, 1.2 equiv) was added. The reaction was left at room temperature for 1h20 and quenched with a saturated solution of NH_4Cl. The product was extracted with Et_2O, washed with brine, dried over $MgSO_4$, filtered and concentrated under reduced pressure. Purification by column chromatography (pentane/Et_2O: 90/10 to 80/20) yielded 261 mg of product **14** as a pure transparent oil (yield: 78%).

[1] *p*-Methoxybenzyl bromide (PMBBr) was prepared from *p*-methoxybenzyl alcohol. This alcohol (10.0 g, 72.36 mmol, 1.0 equiv) was slowly added at 0 °C to a 48% solution of HBr (25 mL, 3.0 equiv) during 15 minutes under strong stirring and further left 15 minutes at 0 °C. After dilution with water and Et_2O at 0 °C, PMBBr was extracted with Et_2O. The extract was washed with brine, dried over $MgSO_4$, filtrated and evaporated under reduced pressure. The purity of PMBBr was checked by NMR before use.

^{1}H NMR (400 MHz, $CDCl_3$) (major stereoisomer) δ 7.31 – 7.23 (m, 2H, ArH), 6.91 – 6.84 (m, 2H, ArH), 5.67 (ddd, J = 17.2, 10.3, 7.6 Hz, 1H, H_8), 4.95 (ddd, J = 17.2, 1.9, 1.2 Hz, 1H, H_{9a}), 4.91 (ddd, J = 10.3, 1.9, 0.9 Hz, 1H, H_{9b}), 4.46 (m, 2H, ArC$\underline{H}_2$), 3.80 (s, 3H, OCH_3), 3.59 – 3.50 (m, 1H, H_4), 3.02 (dddd, J = 6.3, 5.3, 4.0, 2.7 Hz, 1H, H_2), 2.74 (dd, J = 5.0, 4.0 Hz, 1H, H_{1a}), 2.46 (dd, J = 5.0, 2.7 Hz, 1H, H_{1b}), 2.14 – 2.02 (m, 1H, H_7), 1.82 (dt, J = 14.3, 6.3 Hz, 1H, H_{3a}), 1.70 (dt, J = 14.3, 5.3 Hz, 1H, H_{3b}), 1.65 – 1.53 (m, 2H, H_5), 1.45 – 1.29 (m, 2H, H_6), 0.99 (d, J = 6.7 Hz, 3H, 7-CH_3).
^{13}C NMR (101 MHz, $CDCl_3$) (major stereoisomer) δ 159.3 (Ar), 144.6 (C_8), 130.9 (Ar), 129.5 (Ar), 113.9 (Ar), 113.0 (C_9), 76.5 (C_4), 70.6 (Ar$\underline{C}H_2$), 55.4 (OCH_3), 49.8 (C_2), 47.0 (C_1), 37.9 (C_7), 37.0 (C_3), 32.1 (C_6), 31.7 (C_5), 20.4 (7-CH_3).
IR (ATR): $\tilde{\nu}$ =2931, 2860, 1736, 1638, 1611, 1585, 1512, 1455, 1418, 1343, 1300, 1244, 1172, 1070, 1033, 996, 911, 820, 753, 683.

(4*R*,7*S*)-7-Methyl-4-(4-methoxybenzyloxy)nona-8-en-2-one (15)

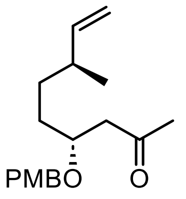
15 (through 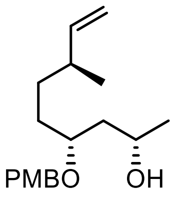 **S3**)

A round bottom flask was charged with 110 mg of **14** (0.38 mmol, 1 equiv) and 4.0 mL of dry THF. The solution was placed at 0 °C and 29 mg of $LiAlH_4$ (0.76 mmol, 2 equiv) was added. The reaction was stirred at 0 °C for 75 min. The reaction was quenched carefully with 1 M NaOH, the product was extracted with Et_2O, washed with brine, dried over $MgSO_4$, filtered and dried under reduced pressure to furnish the crude secondary alcohol **S3** (purification can be performed by column chromatography, petroleum ether/Et_2O: 80/20 to 60/40).
^{1}H NMR (400 MHz, $CDCl_3$) of S3 δ 7.27 – 7.23 (m, 2H, ArH), 6.89 – 6.85 (m, 2H, ArH), 5.68 (ddd, J = 17.2, 10.3, 7.6 Hz, 1H, H_8), 4.97 (ddd, J = 17.2, 1.9, 1.2 Hz, 1H, H_{9a}), 4.94 (ddd, J = 10.3, 1.9, 0.9 Hz, 1H, H_{9b}), 4.57 (d, J = 11.0 Hz, 1H, ArC$\underline{H}_2$-a), 4.35 (d, J = 11.0 Hz, 1H, ArC$\underline{H}_2$-b), 3.98 – 3.88 (m, 1H, H_2), 3.79 (s, 3H, OCH_3), 3.70 – 3.57 (m, 1H, H_4), 2.16 – 2.04 (m, 1H, H_7), 1.72 – 1.48 (m, 4H, H_3 & H_5), 1.41 – 1.23 (m, 2H, H_6), 1.14 (d, J = 6.2 Hz, 3H, H_1), 1.01 (d, J = 6.7 Hz, 3H, 7-CH_3).
The crude extract was solubilized in 4 mL of dry DCM and placed at 0 °C. DMP (177 mg, 0.42 mmol, 1.1 equiv) was then added and the reaction was left at room temperature for 30 min. The reaction was quenched with a saturated solution of

$Na_2S_2O_3$, the product was extracted in DCM, washed with brine, dried over $MgSO_4$, filtered and concentrated under reduced pressure. Purification by column chromatography (petroleum ether/Et_2O: 85/15 to 80/20) yielded 87 mg of product **15** as a transparent oil (yield: 78%).

[α_D]^{25}= − 8.1° (c 0.22, $CHCl_3$).

^{1}H NMR (400 MHz, $CDCl_3$) δ 7.25 – 7.20 (m, 2H, ArH), 6.89 – 6.84 (m, 2H, ArH), 5.66 (ddd, J = 17.2, 10.3, 7.6 Hz, 1H, H_8), 4.95 (ddd, J = 17.2, 1.9, 1.2 Hz, 1H, H_{9a}), 4.91 (ddd, J = 10.3, 1.9, 0.9 Hz, 1H, H_{9b}), 4.45 (d, J = 11.0 Hz, 1H, $ArCH_2$-a), 4.41 (d, J = 11.0 Hz, 1H, $ArCH_2$-b), 3.93 – 3.86 (m, 1H, H_4), 3.79 (s, 3H, OCH_3), 2.74 (dd, J = 15.8, 7.5 Hz, 1H, H_{3a}), 2.48 (dd, J = 15.8, 4.9 Hz, 1H, H_{3b}), 2.15 (s, 3H, H_1), 2.12 – 2.04 (m, 1H, H_7), 1.62 – 1.43 (m, 2H, H_5), 1.40 – 1.28 (m, 2H, H_6), 0.98 (d, J = 6.7 Hz, 3H, 7-CH_3).

^{13}C NMR (101 MHz, $CDCl_3$) δ 207.9 (C_2), 159.3 (Ar), 144.4 (C_8), 130.8 (Ar), 129.5 (Ar), 113.9 (Ar), 113.0 (C_9), 75.4 (C_4), 71.3 (Ar$\underline{C}H_2$), 55.4 (OCH_3), 48.7 (C_3), 37.9 (C_7), 32.0 (C_5), 31.9 (C_6), 31.3 (C_1), 20.4 (7-CH_3).

IR (ATR): $\tilde{\nu}$ = 1712, 1612, 1512, 1355, 1301, 1245, 1172, 1065, 1033, 996, 910, 820, 732, 682, 636.

HRMS (ESI+): Calculated for $C_{16}H_{27}O_3^+$ [M + H^+]: 291.1955; Found: 291.1959.

Ethyl (*R*)-2-oxothiazolidine-4-carboxylate (17)

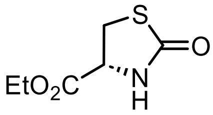

17

A round bottom flask was charged with 2.0 g (10.8 mmol, 1 equiv) of L-cysteine ethyl ester hydrochloride (**16**) and 40 mL of dry DCM. The solution was placed at 0 °C and 1.9 mg of CDI (11.8 mmol, 1.1 equiv) was added. The reaction was stirred at 0 °C for 3 h and quenched with water, the product was extracted with EtOAc and filtered over a tall silica pad, eluting with 3 volumes of EtOAc. After evaporation of the solvent under reduced pressure, 2.4 g of product **17** was isolated as a transparent viscous oil (yield: 85%).

Characterization data were in accordance with those of the literature [5].

[α_D]^{25}= − 51.1 (c 1.95, $CHCl_3$) [Lit. [6] – 51.8 (c 3.14, $CHCl_3$)].

^{1}H NMR (400 MHz, CDCl$_3$) δ 6.29 (b, 1H, NH), 4.42 (ddd, J = 8.2, 5.3, 1.0 Hz, 1H, H_4), 4.27 (q, J = 7.1 Hz, 2H, OC<u>H</u>$_2$CH$_3$), 3.70 (dd, J = 11.4, 8.2 Hz, 1H, H_{5a}), 3.62 (ddd, J = 11.4, 5.3, 0.5 Hz, 1H, H_{5b}), 1.31 (t, J = 7.1 Hz, 3H, OCH$_2$C<u>H</u>$_3$).
^{13}C NMR (101 MHz, CDCl$_3$) δ 174.6 (C_2), 170.0 (<u>C</u>OOEt), 62.6 (O<u>C</u>H$_2$CH$_3$), 56.1 (C_4), 31.9 (C_5), 14.2 (OCH$_2$<u>C</u>H$_3$).

Ethyl (*R*)-*N*-(4-methoxybenzyl)-2-oxothiazolidine-4-carboxylate (18)

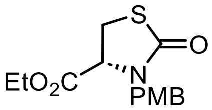

18

A round bottom flask was charged with 1.9 g (11.0 mmol, 1 equiv) of **17** and 40 mL of dry THF. The solution was placed at 0 °C and 438 mg of NaH (60% on mineral oil, 10.9 mmol, 1.0 equiv) was added. The solution was stirred at 0 °C for 5 minutes and 1.73 mL of freshly prepared PMBBr (12.1 mmol, 1.1 equiv) was added. The reaction was left at room temperature overnight and quenched with a saturated solution of NH_4Cl. The product was extracted with EtOAc, washed with brine, dried over $MgSO_4$, filtered and concentrated under reduced pressure. Purification by column chromatography (petroleum ether/EtOAc: 80/20) yielded 2.52 g of product **18** as a transparent oil (yield: 72%).
Characterization data were in accordance with those of the literature [5].
$[\alpha_D]^{25}$= − 92.5 (c 0.87, EtOH) [Lit. [5] – 96.7 (c 1.3, EtOH)].
^{1}H NMR (400 MHz, CDCl$_3$) δ 7.18 – 7.12 (m, 2H, ArH), 6.89 – 6.83 (m, 2H, ArH), 5.08 (d, J = 14.8 Hz, 1H, ArCH$_2$-a), 4.24 (q, J = 7.1 Hz, 2H, OC<u>H</u>$_2$CH$_3$), 4.11 (dd, J = 8.5, 3.1 Hz, 1H, H_4), 3.98 (d, J = 14.8 Hz, 1H, ArCH$_2$-b), 3.79 (s, 3H, OCH$_3$), 3.48 (dd, J = 11.4, 8.5 Hz, 1H, H_{5a}), 3.33 (dd, J = 11.4, 3.1 Hz, 1H, 1H_{5b}), 1.30 (t, J = 7.1 Hz, 3H, OCH$_2$C<u>H</u>$_3$).
^{13}C NMR (101 MHz, CDCl$_3$ δ 171.7 (C_2), 170.1 (<u>C</u>OOEt), 159.5 (Ar), 129.9 (Ar), 127.7 (Ar), 114.4 (Ar), 62.3 (O<u>C</u>H$_2$CH$_3$), 59.4 (C_4), 55.4 (O<u>C</u>H$_3$), 47.4 (Ar<u>C</u>H$_2$), 29.2 (C_5), 14.3 (OCH$_2$<u>C</u>H$_3$).

(*R*)-4-(Hydroxymethyl)-3-(4-methoxybenzyl)thiazolidin-2-one (19)

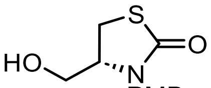

19

A round bottom flask was charged with 2.07 g (7.02 mmol, 1 equiv) of **18**, 40 mL of dry THF and 8 mL of dry EtOH. A first batch of $LiBH_4$ (510 mg, 7.0 mmol, 1.0 equiv) was added and the solution was stirred vigorously for 30 minutes. A second batch of $LiBH_4$ (510 mg, 7.0 mmol, 1.0 equiv) was performed and the solution was stirred vigorously for another 30 minutes. The reaction was carefully quenched with a 1 M HCl solution and the product was extracted with EtOAc, washed with brine, dried over $MgSO_4$, filtered and concentrated under reduced pressure. Purification by column chromatography (petroleum ether/EtOAc: 50/50) yielded 1.60 g of product **19** as a white crystallin solid (yield: 90%). Suitable crystals for X-ray crystallographic analysis were obtained from petroleum ether/ethyl acetate.

Melting point: 74.1°C.

$[\alpha_D]^{25}$= − 9.8 (c 1.14, $CHCl_3$).

^{1}H NMR (400 MHz, $CDCl_3$) δ 7.19-7.24 (m, 2H, ArH), 6.85-6.90 (m, 2H, ArH), 4.74 (d, J = 15.0 Hz, 1H, ArC$\underline{H}_2$-a), 4.26 (d, J = 15.0 Hz, 1H, ArC$\underline{H}_2$-b), 3.88 – 3.70 (m, 5H, OCH_3 & C$\underline{H}_2$OH-a & H_4), 3.64 (ddd, J = 10.4, 6.5, 2.9 Hz, 1H, C$\underline{H}_2$OH-b), 3.30 (dd, J = 11.3, 7.4 Hz, 1H, H_{5a}), 3.26 (dd, J = 11.3, 5.4 Hz, 1H, H_{5b}), 1.50 (dd, J = 6.5, 4.6 Hz, 1H, OH).

^{13}C NMR (101 MHz, $CDCl_3$ δ 173.1 (C_2), 159.3 (Ar), 129.4 (Ar), 128.4 (Ar), 114.4 (Ar), 61.1 ($\underline{C}H_2$OH), 59.6 (C_4), 55.4 (O$\underline{C}H_3$), 46.8 (Ar$\underline{C}H_2$), 28.0 (C_5).

HRMS (ESI+): Calculated for $C_{12}H_{15}NO_3SNa^+$ [M + Na^+]: 276.0665; Found: 276.0664.

(*R*)-3-(4-Methoxybenzyl)-2-oxothiazolidine-4-carbaldehyde (8)

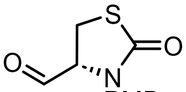

8

A round bottom flask was charged with 400 mg of **19** (1.58 mmol, 1 equiv) and 16 mL of dry DCM. The solution was placed at 0 °C and 737 mg of DMP (1.74 mmol, 1.1 equiv) was added. The reaction was stirred at room temperature for 70 min and quenched with a saturated solution of $Na_2S_2O_3$. The product was extracted with DCM, washed with brine, dried over $MgSO_4$, filtered and concentrated under reduced

pressure. Purification by column chromatography (EP/EtOAc: 50/50) yielded 305 mg of product **8** as a transparent viscous oil (yield: 77%).

$[\alpha_D]^{25}$= − 31.3° (c 0.21, $CHCl_3$).

^{1}H NMR (400 MHz, $CDCl_3$) δ 9.49 (d, J = 1.8 Hz, 1H, CHO), 7.19 – 7.15 (m, 2H, ArH), 6.88 – 6.83 (m, 2H, ArH), 4.85 (d, J = 14.8 Hz, 1H, ArC<u>H</u>$_2$-a), 4.27 (d, J = 14.8 Hz, 1H, ArC<u>H</u>$_2$-b), 4.04 (ddd, J = 9.0, 3.3, 1.8 Hz, 1H, H_4), 3.79 (s, 3H, OCH_3), 3.47 (dd, J = 11.5, 9.0 Hz, 1H, H_{5a}), 3.30 (dd, J = 11.5, 3.3 Hz, 1H, H_{5b}).

^{13}C NMR (101 MHz, $CDCl_3$ δ 197.9 (CHO), 171.7 (C_2), 159.7 (Ar), 130.1 (Ar), 127.3 (Ar), 114.5 (Ar), 65.1 (C_4), 55.4 (OCH_3), 47.9 (Ar<u>C</u>H_2), 26.4 (C_5).

IR (ATR): $\tilde{\nu}$ = 1661, 1610, 1511, 1440, 1393, 1372, 1302, 1244, 1172, 1109, 1028, 978, 840, 815, 663.

HRMS (ESI+): Calculated for $C_{12}H_{14}NO_3S^+$ [M + H^+]: 252.0689; Found: 252.0694.

(*R*)-4-((1*R*,5*R*,8*S*)-1-Hydroxy-5-((4-methoxybenzyl)oxy)-8-methyl-3-oxodec-9-en-1-yl)-3-(4-methoxybenzyl)thiazolidin-2-one (21)

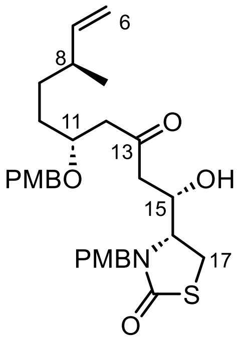

21 *(the atom numbering follows that of latrunculin B, as found in the article)*

A round bottom flask was charged with 356 mg (1.23 mmol, 1 equiv) of ketone **15**, 4 mL of dry DCM, 6 mL of dry Et_2O and 430 µL of DiPEA (2.45 mmol, 2.0 equiv). The solution was placed at −78 °C and 1.35 mL of a 1M solution of Cy_2BCl in hexanes (1.35 mmol, 1.1 equiv) was added slowly. The reaction was stirred at −78 °C for 30 min. The obtained suspension was added dropwise through a syringe transfer at −78 °C into a solution of 343 mg of aldehyde **8** (1.36 mmol, 1.1 equiv) in 4.5 mL of dry DCM. The reaction was kept at −78 °C for 45 min before quenching with a saturated solution of NH_4Cl. The product was extracted with EtOAc, washed with brine, dried over $MgSO_4$, filtered and dried under reduced pressure. The product was purified by column chromatography (EP/EtOAc: 85/15 to 75/25) followed by washing with a saturated solution of $NaHSO_3$. Evaporation of the solvent yielded 343 mg of product **21** as a colorless oil (yield: 55 %) with a dr of 91:9 (NMR).

[α_D]^{25}= − 49.3° (c 0.15, $CHCl_3$).

^{1}H NMR (400 MHz, $CDCl_3$) δ 7.24 – 7.19 (m, 2H, ArH_{OPMB}), 7.19 – 7.14 (m, 2H, ArH_{NPMB}), 6.88 – 6.84 (m, 2H, ArH_{OPMB}), 6.86 – 6.81 (m, 2H, ArH_{NPMB}), 5.66 (ddd, J = 17.2, 10.3, 7.6 Hz, 1H, H_7), 4.96 (ddd, J = 17.2, 1.8, 1.2 Hz, 1H, H_{6a}), 4.94 (ddd, J = 10.3, 1.8, 0.8 Hz, 1H, H_{6b}), 4.78 (d, J = 14.9 Hz, 1H, ArC$\underline{H}_2$O-a), 4.47 (d, J = 11.0 Hz, 1H, ArC$\underline{H}_2$N-a), 4.34 (d, J = 11.0 Hz, 1H, ArC$\underline{H}_2$N-b), 4.36 – 4.30 (m, 1H, H_{15}), 3.96 (d, J = 14.9 Hz, 1H, ArC$\underline{H}_2$O-b), 3.91 – 3.82 (m, 1H, H_{11}), 3.81 – 3.78 (m, 1H, H_{16}), 3.78 (s, 3H, OCH_3), 3.76 (s, 3H, OCH_3), 3.44 (d, J = 3.1 Hz, 1H, OH), 3.24 (dd, J = 11.9, 8.9 Hz, 1H, H_{17a}), 3.06 (dd, J = 11.9, 3.0 Hz, 1H, H_{17b}), 2.64 (dd, J = 15.2, 8.2 Hz, 1H, H_{12a}), 2.59 (s, 1H, H_{14a}), 2.58 (d, J = 2.7 Hz, 1H, H_{14b}), 2.44 (dd, J = 15.2, 4.4 Hz, 1H, H_{12b}), 2.14 – 2.03 (m, 1H, H_8), 1.66 – 1.42 (m, 2H, H_{10}), 1.37 – 1.27 (m, 2H, H_9), 1.00 (d, J = 6.7 Hz, 3H, 8-CH_3).

^{13}C NMR (101 MHz, $CDCl_3$) δ 210.9 (C_{13}), 172.2 (C=$O_{thiazolidinone}$), 159.4 & 159.4 (*para* Ar_{OPMB} and Ar_{NPMB}), 144.3 (C_7), 130.3 (*ipso* Ar_{OPMB}), 129.7 (*ortho* Ar_{NPMB}), 129.6 (*ortho* Ar_{OPMB}), 128.4 (*ipso* Ar_{NPMB}), 114.3 (*meta* Ar_{NPMB}), 114.0 (*meta* Ar_{OPMB}), 113.2 (C_6), 75.5 (C_{11}), 71.1 (Ar$\underline{C}H_2$O), 66.6 (C_{15}), 60.1 (C_{16}), 55.4 & 55.4 (2 OCH_3), 48.3 (C_{12}), 47.3 (Ar$\underline{C}H_2$N), 44.1 (C_{14}), 37.9 (C_8), 31.7 (C_9), 31.5 (C_{10}), 25.7 (C_{17}), 20.4 (8-CH_3).

IR (ATR): $\tilde{\nu}$ = 1667, 1610, 1511, 1398, 1302, 1245, 1216, 1199, 1174, 1108, 1065, 1031, 997, 912, 818.

HRMS (ESI+): Calculated for $C_{30}H_{40}NO_6S^+$ [M + H^+]: 542.2571; Found: 542.2575.

(1*R*,3*R*,5*R*,8*S*)-1-Hydroxy-1-((*R*)-3-(4-methoxybenzyl)-2-oxothiazolidin-4-yl)-5-((4-methoxybenzyl)oxy)-8-methyldec-9-en-3-yl 4-nitrobenzoate (23)

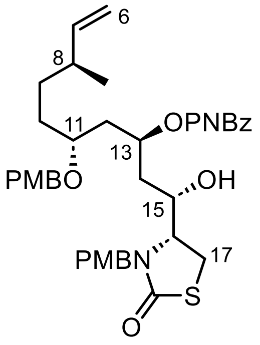

23 (66:10 mixture with **22**)
(the atom numbering follows that of latrunculin B, as found in the article)

A round bottom flask was charged with 67 mg (0.44 mmol, 1.2 equiv) of samarium dust. The argon atmosphere was restored by a cycle of vacuum and argon and 4.4 mL of distilled THF was added. To this suspension was added 30 µL of CH_2I_2 (0.37 mmol,

1 equiv) and the reaction was stirred during 15 min as the solution turns deep blue. This 0.1 M SmI_2 solution was used as such.

Another round bottom flask was charged with 82 mg (0.15 mmol, 1 equiv) of aldol product **21** and 97 mg of *para*-nitrobenzaldehyde (0.45 mmol, 3 equiv). The argon atmosphere was restored by a cycle of vacuum and argon and 1.5 mL of distilled THF was added. After full dissolution, 1.5 mL of the SmI_2 solution (0.15 mmol, 1.0 equiv) was added dropwise and the reaction was left at room temperature for 24 hours. The reaction was quenched with a saturated solution of NH_4Cl. The product was extracted in EtOAc, washed with brine, dried over $MgSO_4$, filtered and dried under reduced pressure. Purification by column chromatography (petroleum ether/EtOAc: 90/10 to 80/20) yielded 79 mg of a 66:10 mixture of unseparable products **23** and **22** as a yellow viscous oil (yield: 76%).

$[\alpha_D]^{25}$= − 51.6 (c 0.32, $CHCl_3$).

^{1}H NMR (400 MHz, $CDCl_3$) of major isomer: δ 8.26 – 8.19 (m, 2H, ArH_{PNB}), 8.10 – 8.02 (m, 2H, ArH_{OPNB}), 7.20 – 7.13 (m, 2H, ArH_{OPMB}), 7.11 – 7.02 (m, 2H, ArH_{NPMB}), 6.80 – 6.75 (m, 2H, ArH_{OPMB}), 6.75 – 6.69 (m, 2H, ArH_{NPMB}), 5.64 (ddd, J = 17.0, 10.5, 7.7 Hz, 1H, H_7), 5.44 – 5.31 (m, 1H, H_{13}), 4.90 (ddd, J = 17.4, 1.8, 1.1 Hz, 1H, H_{6a}) ,4.89 (ddd, J = 10.3, 1.8, 1.0 Hz, 1H, H_{6b}), 4.77 (d, J = 14.9 Hz, 1H, $ArC\underline{H}_2O$-a), 4.50 (d, J = 11.0 Hz, 1H, $ArC\underline{H}_2N$-a), 4.28 (d, J = 11.0 Hz, 1H, $ArC\underline{H}_2N$-b), 4.06 (d, J = 14.9 Hz, 1H, $ArC\underline{H}_2O$-b), 3.79 – 3.76 (m, 2H, H_{15} & H_{16}), 3.74 (s, 3H, OCH_3), 3.73 (s, 3H, OCH_3), 3.63 – 3.58 (m, 1H, OH), 3.57 – 3.51 (m, 1H, H_{11}), 3.23 (dd, J = 11.7, 8.6 Hz, 1H, H_{17a}), 2.99 (dd, J = 11.7, 2.8 Hz, 1H, H_{17b}), 2.16 – 2.01 (m, 2H, H_{12a} & H_8), 1.89 – 1.74 (m, 3H, H_{14} & H_{12b}), 1.63 – 1.50 (m, 2H, H_{10}), 1.38 – 1.27 (m, 2H, H_9), 0.98 (d, J = 6.7 Hz, 3H, 8-CH_3).

^{13}C NMR (101 MHz, $CDCl_3$) of major isomer δ 172.4 ($C{=}O_{thiazolidinone}$), 165.6 ($C{=}O_{PNB}$), 159.3 & 159.3 (*para* Ar_{OPMB} and Ar_{NPMB}), 150.7 (*ipso* Ar_{PNB}), 144.2 (C_7), 135.0 (*para* Ar_{PNB}), 130.9 (*ortho* Ar_{PNB}), 130.1 (*ipso* Ar_{OPMB}), 129.7 (*ortho* Ar_{OPMB}), 129.4 (*ortho* Ar_{NPMB}), 128.3 (*ipso* Ar_{NPMB}), 123.6 (*meta* Ar_{PNB}), 114.2 (*meta* Ar_{NPMB}), 113.8 (*meta* Ar_{OPMB}), 113.2 (C_6), 75.4 (C_{11}), 71.9 (C_{13}), 70.2 ($Ar\underline{C}H_2O$), 66.8 (C_{15}), 61.2 (C_{16}), 55.3 & 55.3 (2 OCH_3), 47.6 ($ArCH_2N$), 38.9 (C_{12}), 37.9 (C_8), 36.0 (C_{14}), 31.4 (C_9), 30.9 (C_{10}), 26.0 (C_{17}), 20.4 (8-CH_3).

IR (ATR): $\tilde{\nu}$ = 1665, 1526, 1512, 1347, 1272, 1246, 1174, 1102, 1032, 1014, 908, 819, 720, 665.

HRMS (ESI+): Calculated for $C_{37}H_{45}N_2O_9S^+$ [M + H$^+$]: 693.2840; Found: 693.2831.

Data for minor isomer 22

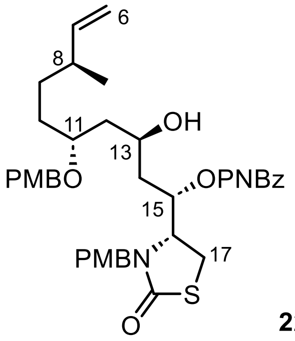

22 *(the atom numbering follows that of latrunculin B, as found in the article)*

Compound **22** was obtained during optimization attempts, through a slightly different, but poorly reproducible method: a round bottom flask was charged with 17 mg (0.031 mmol, 1 equiv) of **21** and 24 mg of *para*-nitrobenzaldehyde (0.157 mmol, 5 equiv). An argon atmosphere was restored by a cycle of vacuum and argon and 0.3 mL of distilled THF was added. The reaction was placed at 0 °C and 30 µL of an SmI_2 solution freshly prepared as above (0.003 mmol, 10% equiv) was added. The reaction was left at room temperature for 5 h. The reaction was quenched with a saturated solution of NH_4Cl. The product was extracted in EtOAc, washed with brine, dried over $MgSO_4$, filtered and dried under reduced pressure. Purification by column chromatography (petroleum ether/EtOAc: 85/15 to 80/20) yielded 16 mg of product **22** (yield: 71%) containing residual amounts of starting material **21**.

^{1}H NMR (400 MHz, $CDCl_3$) δ 8.30 – 8.26 (m, 2H, *meta* ArH_{PNB}), 8.19 – 8.13 (m, 2H, *ortho* ArH_{PNB}), 7.35 – 7.29 (m, 2H, *ortho* ArH_{NPMB}), 7.19 – 7.16 (m, 2H, *ortho* ArH_{OPMB}), 6.92 – 6.86 (m, 2H, *meta* ArH_{NPMB}), 6.77 – 6.74 (m, 2H, *meta* ArH_{OPMB}), 5.82 (ddd, J = 10.6, 4.0, 2.0 Hz, 1H, H_{15}), 5.65 (ddd, J = 17.2, 10.3, 7.6 Hz, 1H, H_7), 5.07 (d, J = 14.9 Hz, 1H, $ArC\underline{H}_2O$), 4.95 (ddd, J = 17.2, 1.9, 1.2 Hz, 1H, H_{6a}), 4.92 (ddd, J = 10.3, 1.9, 0.9 Hz, 1H, H_{6b}), 4.54 (d, J = 11.1 Hz, 1H, $ArC\underline{H}_2N$), 4.30 (d, J = 11.1 Hz, 1H, $ArC\underline{H}_2N$), 4.06 – 4.01 (m, 1H, H_{16}), 4.02 (d, J = 14.9 Hz, 1H, $ArC\underline{H}_2O$), 3.79 (s, 3H, OCH_3), 3.78 – 3.76 (m, 1H, H_{13}), 3.75 (s, 3H, OCH_3), 3.71 (br s, 1H, OH), 3.67 – 3.58 (m, 1H, H_{11}), 3.30 (dd, J = 11.6, 8.8 Hz, 1H, H_{17a}), 3.21 (dd, J = 11.6, 3.3 Hz, 1H, H_{17b}), 2.15 – 2.03 (m, 1H, H_8), 1.97 – 1.78 (m, 2H, H_{14}), 1.78 – 1.68 (m, 1H, H_{12a}), 1.66 – 1.48 (m, 3H, H_{12b} & H_{10}), 1.39 – 1.27 (m, 2H, H_9), 0.99 (d, J = 6.7 Hz, 3H, 8-CH_3).

^{13}C NMR (101 MHz, $CDCl_3$) δ 172.0 ($C{=}O_{thiazolidinone}$), 164.0 ($C{=}O_{PNB}$), 159.6 & 159.4 (2 *para* Ar_{PMB}), 150.9 (*para* Ar_{PNB}), 144.3 (C_7), 135.1 (*ipso* Ar_{PNB}), 130.9 (*ortho* Ar_{PNB}), 130.1 (*ortho* Ar_{NPMB}), 130.0 (*ipso* Ar_{OPMB}), 129.5 (*ortho* Ar_{OPMB}), 127.9 (*ipso* Ar_{NPMB}), 123.8 (*meta* Ar_{PNB}), 114.4 (*meta* Ar_{NPMB}), 114.0 (*meta* Ar_{OPMB}), 113.2 (C_6), 79.1 (C_{11}),

70.7 (C_{15}), 69.9 (Ar<u>C</u>H_2O), 67.3 (C_{13}), 57.6 (C_{16}), 55.4 & 55.3 (2 OCH_3), 46.9 (Ar<u>C</u>H_2N), 41.4 (C_{12}), 38.0 (C_8), 35.9 (C_{14}), 31.2 (C_9), 30.8 (C_{10}), 26.1 (C_{17}), 20.4 (8-CH_3).

(*R*)-4-((1*S*,3*R*,5*R*,8*S*)-1,3-Dihydroxy-5-((4-methoxy-benzyl)oxy)-8-methyldec-9-en-1-yl)-3-(4-methoxybenzyl)thiazolidin-2-one (24)

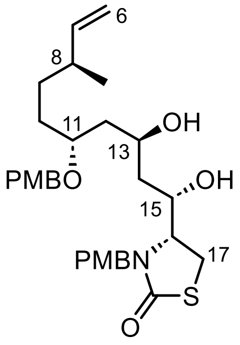

24 *(the atom numbering follows that of latrunculin B, as found in the article)*

A round bottom flask was charged with 17 mg (0.025 mmol, 1 equiv) of unseparable **22**/**23** and 0.4 mL of MeOH. NaOH (0.120 mmol, 4.8 equiv) was added. The reaction was left at room temperature for 30 min and then quenched with a solution of NaOH 1 M. The product was extracted in EtOAc, washed with brine, dried over $MgSO_4$, filtered and dried under reduced pressure. Purification by column chromatography (petroleum ether/EtOAc: 75/25 to 60/40) yielded 13 mg of pure diol product **24** as a light-yellow viscous oil (yield: 97%).

^{1}H NMR (400 MHz, $CDCl_3$) δ 7.25 – 7.19 (m, 4H, *ortho* Ar_{OPMB} and Ar_{NPMB}), 6.91 – 6.82 (m, 4H, *meta* Ar_{OPMB} and Ar_{NPMB}), 5.71 (ddd, J = 17.2, 10.3, 7.6 Hz, 1H, H_7), 5.01 (ddd, J = 14.3, 1.8, 0.9 Hz, 1H, H_{6a}), 4.97 (ddd, J = 7.5, 1.8, 0.9 Hz, 1H, H_{6b}), 4.80 (d, J = 14.8 Hz, 1H, ArC<u>H</u>$_2$N-a), 4.60 (d, J = 10.8 Hz, 1H, ArC<u>H</u>$_2$O-a), 4.36 (brs, 1H, OH), 4.31 (d, J = 10.8 Hz, 1H, ArC<u>H</u>$_2$O-b), 4.27 – 4.21 (m, 1H, H_{15}), 4.20 (d, J = 14.8 Hz, 1H, ArC<u>H</u>$_2$N-b), 4.13 – 4.07 (m, 1H, H_{13}), 3.96 (brs, 1H, OH), 3.80 (s, 3H, OCH_3), 3.78 (s, 3H, OCH_3), 3.72 – 3.65 (m, 1H, H_{11}), 3.29 (dd, J = 11.7, 8.9 Hz, 1H, H_{17a}), 3.19 (dd, J = 11.7, 3.1 Hz, 1H, H_{17b}), 2.17 – 2.08 (m, 1H, H_8), 1.88 – 1.77 (m, 1H, H_{14}), 1.77 – 1.66 (m, 1H, H_{12}), 1.65 – 1.54 (m, 2H, H_9 & H_{10}), 1.48 (ddd, J = 14.4, 5.4, 1.9 Hz, 1H, H_{14}), 1.39 – 1.25 (m, 3H, H_{12} & H_{10} & H_9), 1.04 (d, J = 6.7 Hz, 3H, 8-CH_3).

^{13}C NMR (101 MHz, $CDCl_3$) δ 172.6 (C=$O_{thiazolidinone}$), 159.6 & 159.4 (2 *para* Ar_{PMB}), 144.3 (C_7), 129.8 (2 *ortho* Ar_{PMB}), 129.6 (*ipso* Ar_{NPMB}), 128.9 (*ipso* Ar_{OPMB}), 114.3 & 114.2 (*meta* Ar_{PMB}), 113.3 (C_6), 80.4 (C_{11}), 71.0 (C_{13}), 70.4 (Ar<u>C</u>H_2O), 68.0 (C_{15}), 61.4 (C_{16}), 55.4 (2 OCH_3), 47.7 (Ar<u>C</u>H_2N), 39.2 (C_{12}), 38.2 (C_8), 35.5 (C_{14}), 31.0 (C_9 or C_{10}), 30.7 (C_9 or C_{10}), 26.0 (C_{17}), 20.4 (8-CH_3).

[α$_D$]^{25}= −43.2 (c 0.04, $CHCl_3$).

(*R*)-4-((1*S*,3*R*,5*R*,8*S*)-1,3-((*R*)-(4-Methoxyphenyl)methylenedioxy)-5-((4-methoxy-benzyl)oxy)-8-methyldec-9-en-1-yl)-3-(4-methoxybenzyl)thiazolidin-2-one (25)

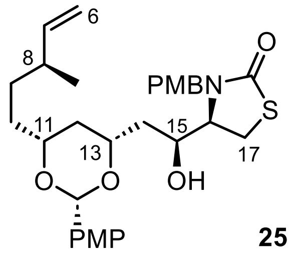

25 *(the atom numbering follows that of latrunculin B, as found in the article)*

A round bottom flask was charged with 5 mg (0.0092 mmol, 1 equiv) of **24**, 4 Å molecular sieves and 0.2 mL of dry DCM. The reaction mixture was placed at 0 °C and a first solution of DDQ (0.0119 mmol, 1.3 equiv) in 0.6 mL of dry DCM with 4 Å molecular sieves was added. The reaction was left at 0 °C for 3 h. A second solution of DDQ (0.0088 mmol, 0.9 equiv) in 0.5 mL of dry DCM with 4 Å molecular sieves was added. The reaction was left at 0 °C for 40 min and then at room temperature for 3 h. The reaction was then filtered and quenched with a saturated solution of $NaHCO_3$. The product was extracted in EtOAc, washed with brine, dried over $MgSO_4$, filtered and dried under reduced pressure. Purification by column chromatography (petroleum ether/EtOAc: 80/20 to 50/50) yielded 4 mg of pure ketal product **27** as a light-yellow viscous oil (yield: 74%).

^{1}H NMR (400 MHz, $CDCl_3$) δ 7.41 – 7.34 (m, 2H, *ortho* Ar_{PMP}), 7.21 – 7.12 (m, 2H, *ortho* Ar_{NPMB}), 6.92 – 6.86 (m, 2H, *meta* Ar_{PMP}), 6.86 – 6.80 (m, 2H, *meta* Ar_{NPMB}), 5.69 (ddd, J = 17.2, 10.3, 7.6 Hz, 1H, H_7), 5.43 (s, 1H, $ArC\underline{H}O_2$), 5.02 – 4.88 (m, 2H, H_6), 4.80 (d, J = 14.9 Hz, 1H, $ArC\underline{H}_2N$-a), 4.34 – 4.25 (m, 1H, H_{15}), 4.20 (d, J = 14.9 Hz, 1H, $ArC\underline{H}_2N$-b), 4.17 – 4.08 (m, 1H, H_{13}), 3.83 – 3.76 (m, 1H, H_{16}), 3.80 (s, 3H, OCH_3), 3.79 (s, 3H, OCH_3), 3.76 – 3.71 (m, 1H, H_{11}), 3.31 (dd, J = 11.7, 8.8 Hz, 1H, H_{17a}), 3.21 (dd, J = 11.7, 3.6 Hz, 1H, H_{17b}), 2.16 – 2.09 (m, 1H, H_8), 1.91 (ddd, J = 14.4, 10.5, 3.7 Hz, 1H, H_{14}), 1.72 – 1.57 (m, 2H, H_{14} & H_{10}), 1.53 – 1.44 (m, 2H, H_{10} & H_9), 1,42 – 1,27 (m, 3H, H_{12} & H_9), 1.02 (d, J = 6.7 Hz, 3H, 8-CH_3).

^{13}C NMR (101 MHz, $CDCl_3$) δ 172.7 ($C{=}O_{thiazolidinone}$), 160.1 (*para* Ar_{PMP}), 159.4 (*para* Ar_{PMB}), 144.5 (C_7), 131.0 (*ipso* Ar_{PMP}), 129.7 (*ortho* Ar_{NPMB}), 128.7 (*ipso* Ar_{NPMB}), 127.4 (*ortho* Ar_{PMP}), 114.3 (*meta* Ar_{NPMB}), 113.8 (*meta* Ar_{PMP}), 113.1 (C_6), 101.2 (C_{acetal}), 77.2 (C_{11}), 74.6 (C_{13}), 67.3 (C_{15}), 61.4 (C_{16}), 55.5 & 55.4 (2 OCH_3), 47.7 ($Ar\underline{C}H_2N$), 37.9 (C_8), 35.6 (C_{12}), 35.2 (C_{14}), 33.6 (C_{10}), 31.9 (C_9), 26.1 (C_{17}), 20.4 (8-CH_3).

$[\alpha_D]^{25}$= − 15.1 (c 0.05, $CHCl_3$).

Crystallographic data for compound 19

The picture of the compound structures shown in the article was generated from Mercury 2022.1.0. The crystal structures of **19** was deposited on the Cambridge Crystallographic Data Centre under CCDC number 2225628.

Single crystals of $C_{12}H_{15}NO_3S$ (**19**) were crystallized from petroleum ether/ethyl acetate. A suitable crystal was selected and mounted on a Bruker APEX-II CCD diffractometer. The crystal was kept at 150.0 K during data collection. Using Olex2 [7], the structure was solved with the SHELXT [8] structure solution program using Intrinsic Phasing and refined with the SHELXL [9] refinement package using Least Squares minimisation.

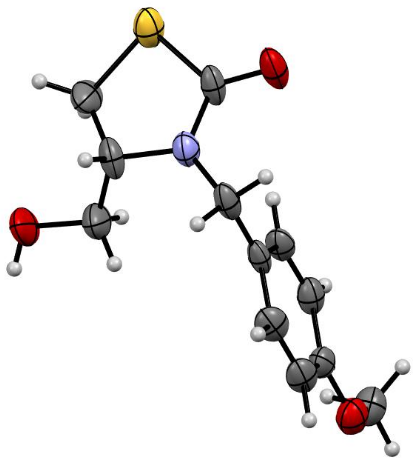

X-ray crystallographic structure of 19

Table S2. Crystal data and structure refinement for **19**.

Identification code	BJm04_0m
Empirical formula	$C_{12}H_{15}NO_3S$
Formula weight	253.31
Temperature/K	150.0
Crystal system	monoclinic
Space group	$P2_1$
a/Å	5.5075(16)

b/Å	5.6395(15)
c/Å	19.524(6)
α/°	90
β/°	93.936(9)
γ/°	90
Volume/$Å^3$	605.0(3)
Z	2
$\rho_{calc}g/cm^3$	1.391
μ/mm^{-1}	0.263
F(000)	268.0
Crystal size/mm^3	0.35 × 0.3 × 0.12
Radiation	MoKα (λ = 0.71073)
2Θ range for data collection/°	4.182 to 54.916
Index ranges	-7 ≤ h ≤ 6, -7 ≤ k ≤ 7, -25 ≤ l ≤ 25
Reflections collected	9492
Independent reflections	2769 [R_{int} = 0.0876, R_{sigma} = 0.0987]
Data/restraints/parameters	2769/9/167
Goodness-of-fit on F^2	1.078
Final R indexes [I>=2σ (I)]	R_1 = 0.0832, wR_2 = 0.2167
Final R indexes [all data]	R_1 = 0.1064, wR_2 = 0.2346
Largest diff. peak/hole / e $Å^{-3}$	1.17/-0.51
Flack parameter	0.0(3)

Table S3. Fractional Atomic Coordinates (×10^4) and Equivalent Isotropic Displacement Parameters ($Å^2$×10^3) for **19**. U_{eq} is defined as 1/3 of of the trace of the orthogonalised U_{IJ} tensor.

**Atom**	***x***	***y***	***z***	**U(eq)**
C1	701(12)	2974(11)	8536(3)	39.3(14)
C3	3088(12)	6466(13)	8740(3)	41.8(16)
C4	5543(16)	7022(18)	8495(4)	64(3)
C5	1007(12)	5618(11)	7567(3)	40.7(16)

Table S3. Fractional Atomic Coordinates (×10^4) and Equivalent Isotropic Displacement Parameters (Å^2×10^3) for **19**. U_{eq} is defined as 1/3 of of the trace of the orthogonalised U_{IJ} tensor.

**Atom**	***x***	***y***	***z***	**U(eq)**
C6	2795(12)	5012(10)	7051(3)	33.8(14)
C7	4301(12)	3002(12)	7113(3)	39.9(15)
C8	5880(12)	2453(12)	6625(3)	39.7(15)
C9	6060(13)	3879(13)	6050(3)	39.5(15)
C10	4565(13)	5882(12)	5982(3)	41.9(17)
C11	3011(13)	6399(14)	6469(3)	43.4(16)
C12	9227(15)	1513(16)	5612(4)	52.6(19)
N1	1689(10)	4920(10)	8267(3)	40.5(13)
O1	-604(10)	1551(9)	8212(3)	52.5(14)
O2	6823(11)	8484(13)	8975(3)	72(2)
O3	7551(9)	3474(9)	5541(2)	48.0(13)
S1	1459(3)	2760(3)	9424.4(8)	48.6(6)
C2A	3690(30)	5070(30)	9407(6)	52(4)
C2	2650(60)	5770(40)	9463(10)	51(4)

Table S4. Anisotropic Displacement Parameters (Å^2×10^3) for **19**. The Anisotropic displacement factor exponent takes the form: $-2\pi^2[h^2a^{*2}U_{11}+2hka^*b^*U_{12}+\ldots]$.

**Atom**	**U_{11}**	**U_{22}**	**U_{33}**	**U_{23}**	**U_{13}**	**U_{12}**
C1	43(3)	23(3)	48(3)	-4(3)	-19(3)	2(3)
C3	40(4)	29(3)	54(4)	-12(3)	-15(3)	0(3)
C4	66(5)	80(6)	44(4)	-14(4)	2(4)	-37(5)
C5	46(4)	28(3)	45(4)	3(3)	-20(3)	-6(3)
C6	41(3)	17(3)	40(3)	1(3)	-14(3)	-4(3)
C7	54(4)	27(3)	37(3)	7(3)	-11(3)	-5(3)
C8	47(4)	29(3)	41(3)	-1(3)	-14(3)	2(3)
C9	47(4)	41(4)	29(3)	0(3)	-7(3)	-10(3)
C10	51(4)	36(4)	37(3)	10(3)	-17(3)	-1(3)

Table S4. Anisotropic Displacement Parameters ($Å^2 \times 10^3$) for **19**. The Anisotropic displacement factor exponent takes the form: $-2\pi^2[h^2a^{*2}U_{11}+2hka^*b^*U_{12}+\ldots]$.

**Atom**	**U_{11}**	**U_{22}**	**U_{33}**	**U_{23}**	**U_{13}**	**U_{12}**
C11	45(4)	40(4)	43(4)	12(3)	-15(3)	1(3)
C12	57(5)	55(5)	45(4)	-9(4)	0(4)	-1(4)
N1	44(3)	36(3)	39(3)	-4(2)	-8(2)	-6(3)
O1	64(3)	32(3)	58(3)	1(2)	-24(3)	-12(3)
O2	77(4)	89(5)	48(3)	-5(3)	-14(3)	-52(4)
O3	55(3)	47(3)	40(2)	0(2)	-8(2)	-1(2)
S1	58.1(11)	38.4(9)	46.1(9)	1.3(8)	-18.3(8)	0.7(9)
C2A	35(8)	76(9)	45(5)	-8(6)	3(6)	-22(7)
C2	34(9)	74(10)	46(6)	-15(7)	1(7)	-25(8)

Table S5. Bond Lengths for **19**.

**Atom**	**Atom**	**Length/Å**	**Atom**	**Atom**	**Length/Å**
C1	N1	1.348(9)	C6	C7	1.405(9)
C1	O1	1.225(8)	C6	C11	1.390(9)
C1	S1	1.759(6)	C7	C8	1.369(9)
C3	C4	1.498(11)	C8	C9	1.391(9)
C3	N1	1.452(8)	C9	C10	1.399(10)
C3	C2A	1.540(14)	C9	O3	1.351(8)
C3	C2	1.500(19)	C10	C11	1.355(10)
C4	O2	1.402(9)	C12	O3	1.441(10)
C5	C6	1.496(9)	S1	C2A	1.790(12)
C5	N1	1.446(8)	S1	C2	1.82(2)

Table S6. Bond Angles for **19**.

**Atom**	**Atom**	**Atom**	**Angle/˚**	**Atom**	**Atom**	**Atom**	**Angle/˚**
N1	C1	S1	111.5(5)	C7	C8	C9	121.0(6)
O1	C1	N1	124.7(6)	C8	C9	C10	118.0(6)
O1	C1	S1	123.8(5)	O3	C9	C8	125.2(7)

Table S6. Bond Angles for **19**.

Atom	Atom	Atom	Angle/˚	Atom	Atom	Atom	Angle/˚
C4	C3	C2A	103.4(8)	O3	C9	C10	116.7(6)
C4	C3	C2	124.2(14)	C11	C10	C9	120.2(6)
N1	C3	C4	111.9(6)	C10	C11	C6	123.1(7)
N1	C3	C2A	107.7(7)	C1	N1	C3	116.8(6)
N1	C3	C2	109.3(10)	C1	N1	C5	120.3(6)
O2	C4	C3	109.4(6)	C5	N1	C3	122.0(6)
N1	C5	C6	115.7(6)	C9	O3	C12	118.4(6)
C7	C6	C5	122.5(5)	C1	S1	C2A	92.8(4)
C11	C6	C5	121.2(6)	C1	S1	C2	92.1(7)
C11	C6	C7	116.3(6)	C3	C2A	S1	106.3(7)
C8	C7	C6	121.4(6)	C3	C2	S1	106.5(12)

Table S7. Torsion Angles for **19**.

A	B	C	D	Angle/˚	A	B	C	D	Angle/˚
C1	S1	C2A	C3	19.3(11)	N1	C1	S1	C2	11.6(12)
C1	S1	C2	C3	-17.6(19)	N1	C3	C4	O2	-179.1(7)
C4	C3	N1	C1	129.3(8)	N1	C3	C2A	S1	-22.6(13)
C4	C3	N1	C5	-61.5(9)	N1	C3	C2	S1	19(2)
C4	C3	C2A	S1	-141.3(9)	N1	C5	C6	C7	31.4(8)
C4	C3	C2	S1	-116.4(14)	N1	C5	C6	C11	-150.1(6)
C5	C6	C7	C8	178.4(6)	O1	C1	N1	C3	176.9(7)
C5	C6	C11	C10	-178.4(6)	O1	C1	N1	C5	7.5(10)
C6	C5	N1	C1	-101.9(7)	O1	C1	S1	C2A	170.3(9)
C6	C5	N1	C3	89.3(7)	O1	C1	S1	C2	-167.0(13)
C6	C7	C8	C9	0.4(9)	O3	C9	C10	C11	179.6(6)
C7	C6	C11	C10	0.1(10)	S1	C1	N1	C3	-1.6(7)
C7	C8	C9	C10	-0.5(10)	S1	C1	N1	C5	-171.0(5)
C7	C8	C9	O3	-179.6(6)	C2A	C3	C4	O2	-63.4(11)
C8	C9	C10	C11	0.5(10)	C2A	C3	N1	C1	16.3(11)

Table S7. Torsion Angles for **19**.

A	B	C	D	Angle/˚	A	B	C	D	Angle/˚
C8	C9	O3	C12	-4.7(9)	C2A	C3	N1	C5	-174.5(10)
C9	C10	C11	C6	-0.3(10)	C2	C3	C4	O2	-44.3(16)
C10	C9	O3	C12	176.2(6)	C2	C3	N1	C1	-12.2(17)
C11	C6	C7	C8	-0.2(9)	C2	C3	N1	C5	157.0(16)
N1	C1	S1	C2A	-11.1(8)					

Table S8. Hydrogen Atom Coordinates (Å×10^4) and Isotropic Displacement Parameters (Å^2×10^3) for **19**.

Atom	*x*	*y*	*z*	U(eq)
H3A	2167.7	7948.45	8830.49	50
H3B	2210.22	8012.99	8683.73	50
H4A	6460.84	5534.85	8435.73	76
H4B	5354.7	7835.61	8044.66	76
H5A	746.01	7354.61	7557.25	49
H5B	-566.63	4858.02	7423.43	49
H7	4220.78	2001.58	7502.87	48
H8	6868.76	1076.85	6680.63	48
H10	4640.06	6882.38	5592.38	50
H11	2022.14	7774.58	6411.19	52
H12A	10162.34	1415.7	5203.97	79
H12B	8316.75	36	5661.45	79
H12C	10340.15	1752.33	6019.75	79
H2	7878.15	9259.02	8779.18	108
H2AA	3607.38	6114.3	9811.43	62
H2AB	5345.32	4381.73	9410.26	62
H2A	1464.94	6863.47	9655.35	62
H2B	4190.29	5836.85	9755.86	62

Table S9. Atomic Occupancy for **19**.

**Atom**	***Occupancy***	**Atom**	***Occupancy***	**Atom**	***Occupancy***
H3A	0.65(3)	H3B	0.35(3)	C2A	0.65(3)
H2AA	0.65(3)	H2AB	0.65(3)	C2	0.35(3)
H2A	0.35(3)	H2B	0.35(3)		

References

(1) Paquette, L. A.; Maynard, G. D. *J. Am. Chem. Soc.* **1992**, *114*, 5018–5027. doi:10.1021/ja00039a011

(2) Magauer, T.; Martin, H. J.; Mulzer, J. *Angew. Chem. Int. Ed. Engl.* **2009**, *48*, 6032–6036. doi:10.1002/anie.200900522

(3) Hoye, T. R.; Jeffrey, C. S.; Shao, F. *Nat Protoc* **2007**, *2*, 2451–2458. doi:10.1038/nprot.2007.354

(4) Mihelich, E. D.; Daniels, K.; Eickhoff, D. J. *J. Am. Chem. Soc.* **1981**, *103*, 7690–7692. doi:10.1021/ja00415a067

(5) Fürstner, A.; De Souza, D.; Turet, L.; Fenster, M. D. B.; Parra-Rapado, L.; Wirtz, C.; Mynott, R.; Lehmann, C. W. *Chem. Eur. J.* **2007**, *13*, 115–134. doi:10.1002/chem.200601135

(6) Fürstner, A.; Kirk, D.; Fenster, M. D. B.; Aïssa, C.; De Souza, D.; Nevado, C.; Tuttle, T.; Thiel, W.; Müller, O. *Chem. Eur. J.* **2007**, *13*, 135–149. doi:10.1002/chem.200601136

(7) Dolomanov, O. V.; Bourhis, L. J.; Gildea, R. J.; Howard, J. a. K.; Puschmann, H. *J. Appl. Cryst.* **2009**, *42*, 339–341. doi:10.1107/S0021889808042726

(8) Sheldrick, G. M. *Acta Cryst. A* **2015**, *71*, 3–8. doi:10.1107/S2053273314026370

(9) Sheldrick, G. M. *Acta Cryst. C* **2015**, *71*, 3–8. doi:10.1107/S2053229614024218

Copies of NMR spectra

(*S*)-4-Methylhex-5-en-1-ol (11)

^{1}H NMR (400 MHz, $CDCl_3$)

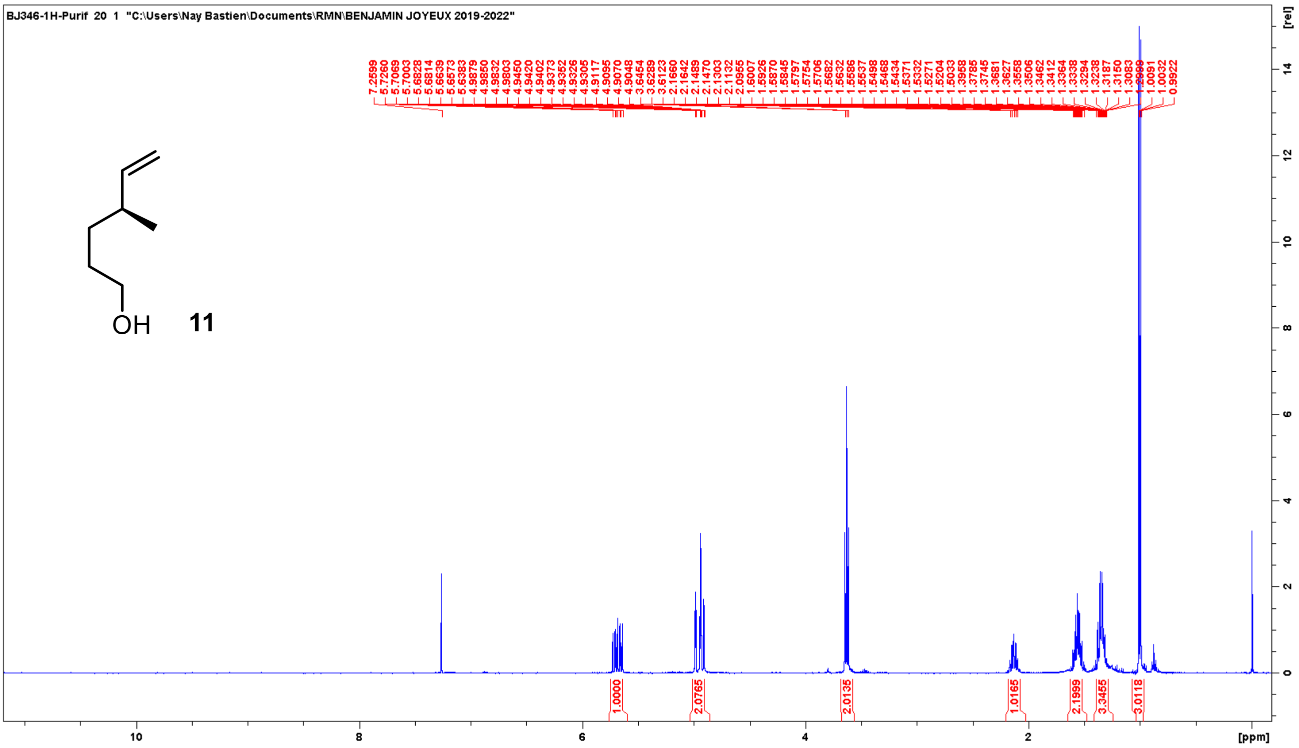

^{13}C NMR (100 MHz, $CDCl_3$)

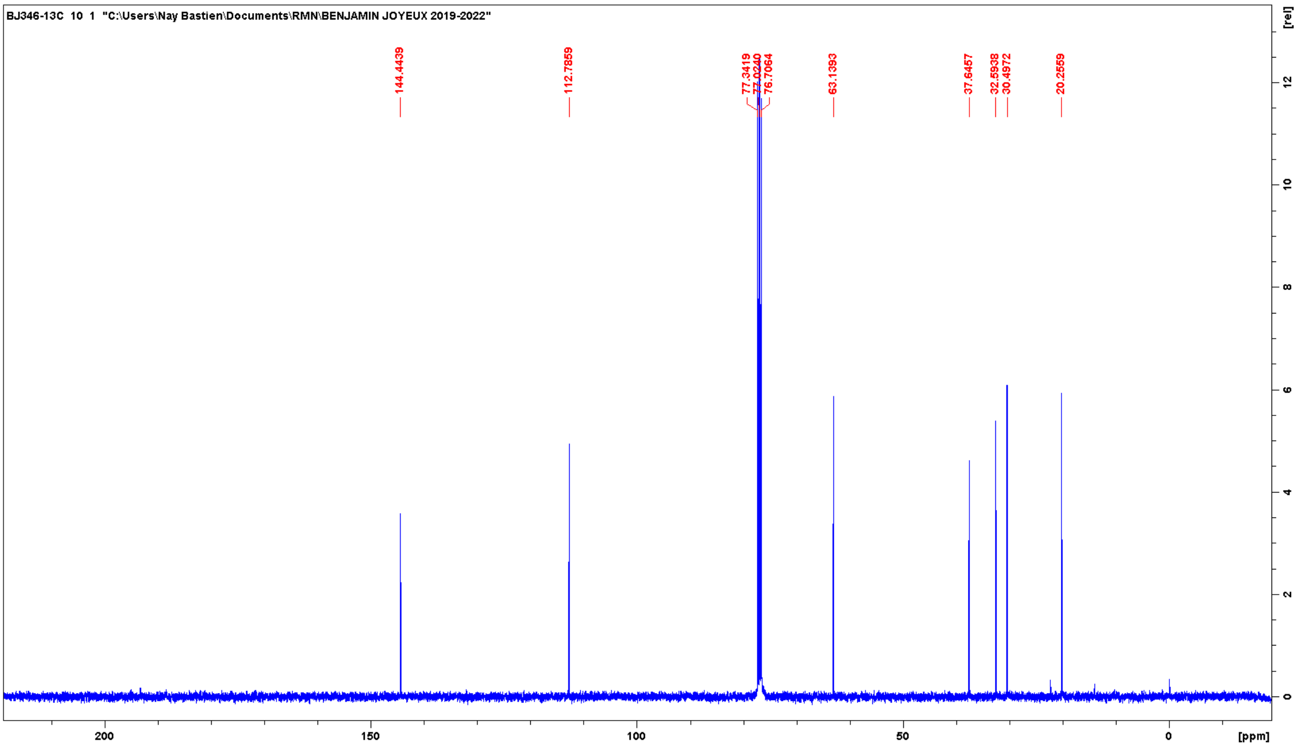

(4*R*,7*S*)-7-Methylnona-1,8-dien-4-ol (12)

^{1}H NMR (400 MHz, $CDCl_3$)

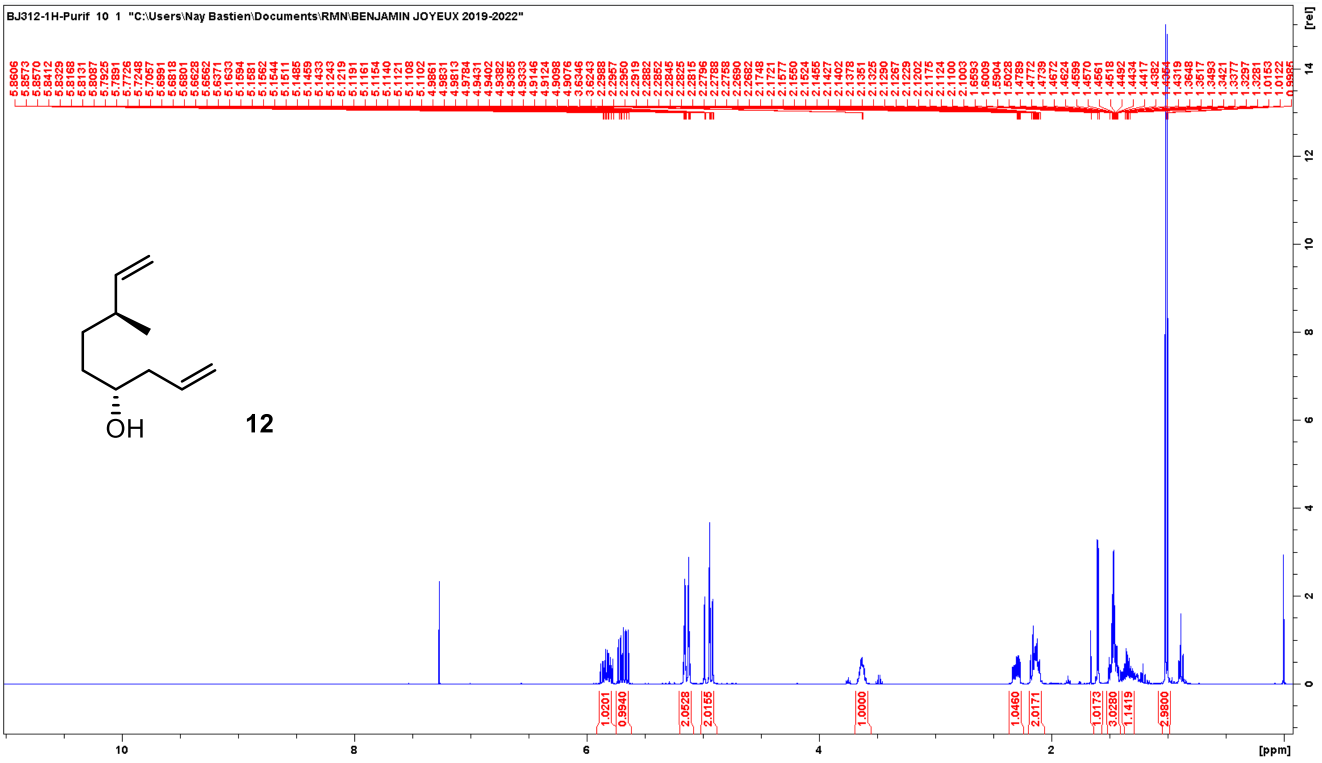

12

^{13}C NMR (100 MHz, $CDCl_3$)

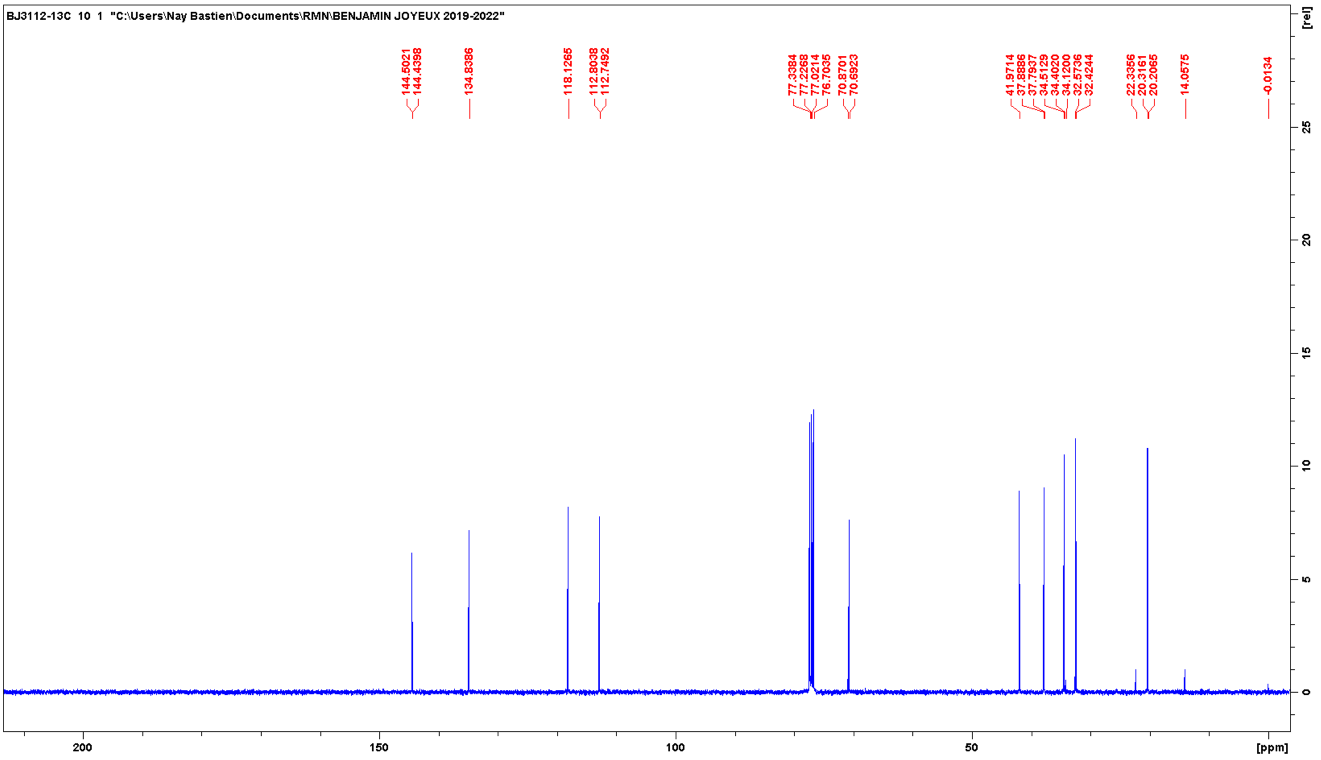

(2*R*,4*R*,7*S*)-1,2-Epoxy-7-methylnona-8-en-4-ol (13)

^{1}H NMR (400 MHz, $CDCl_3$)

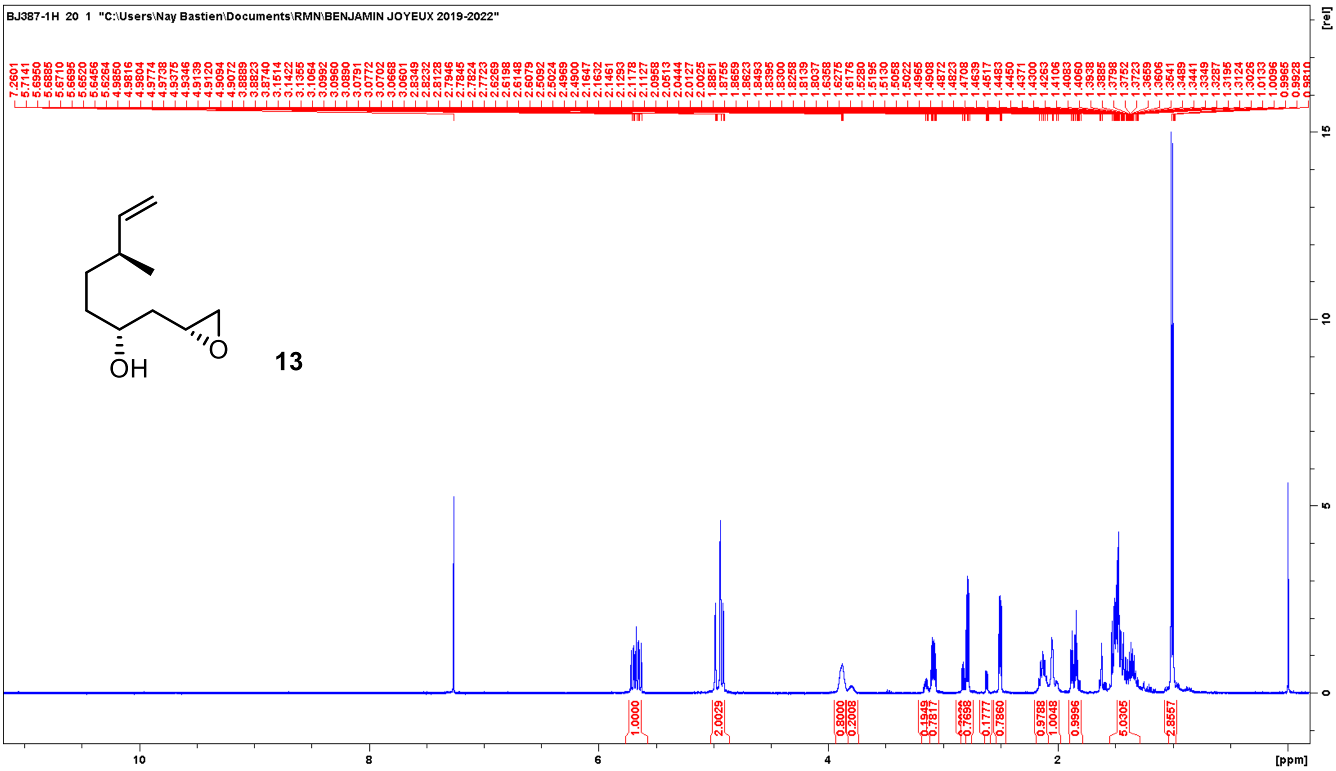

^{13}C NMR (100 MHz, $CDCl_3$)

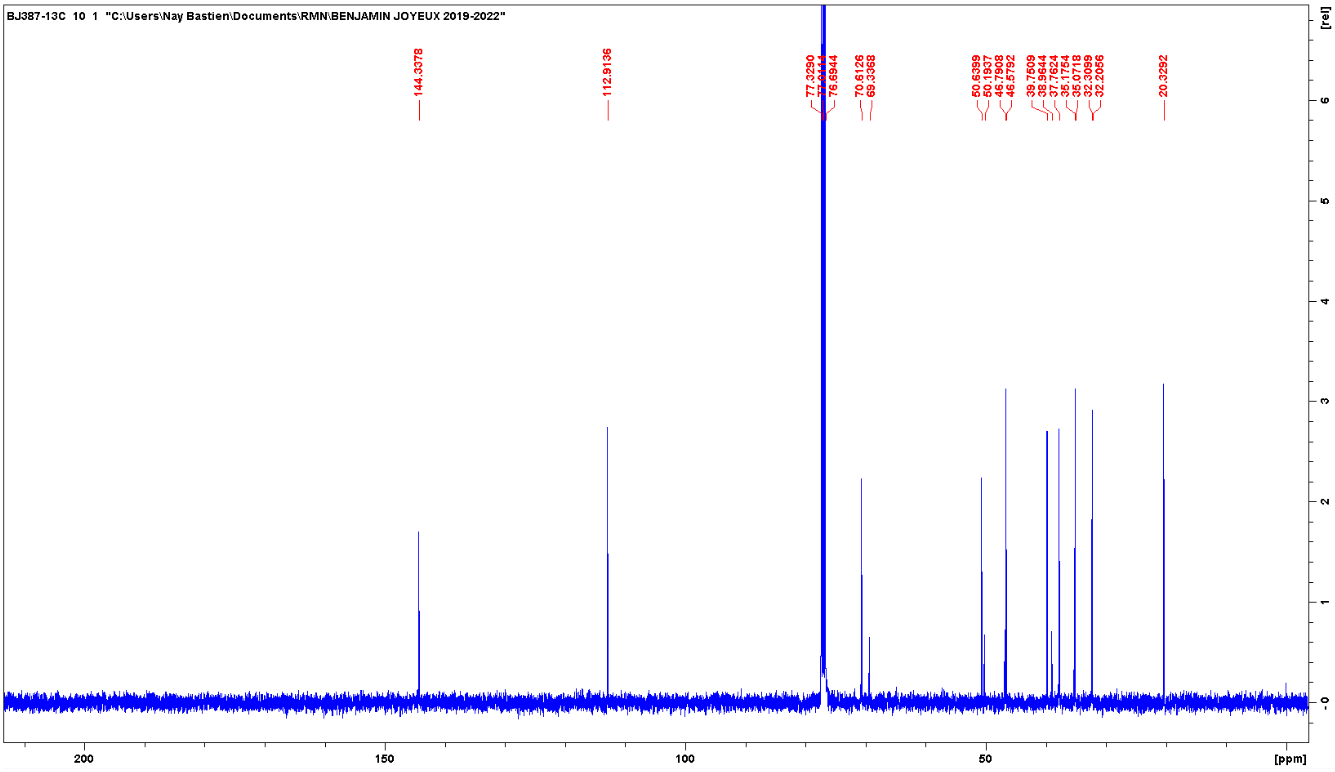

(2*R*,4*R*,7*S*)-1,2-Epoxy-7-methyl-4-(4-methoxybenzyloxy)nona-8-ene (14)

^{1}H NMR (400 MHz, $CDCl_3$)

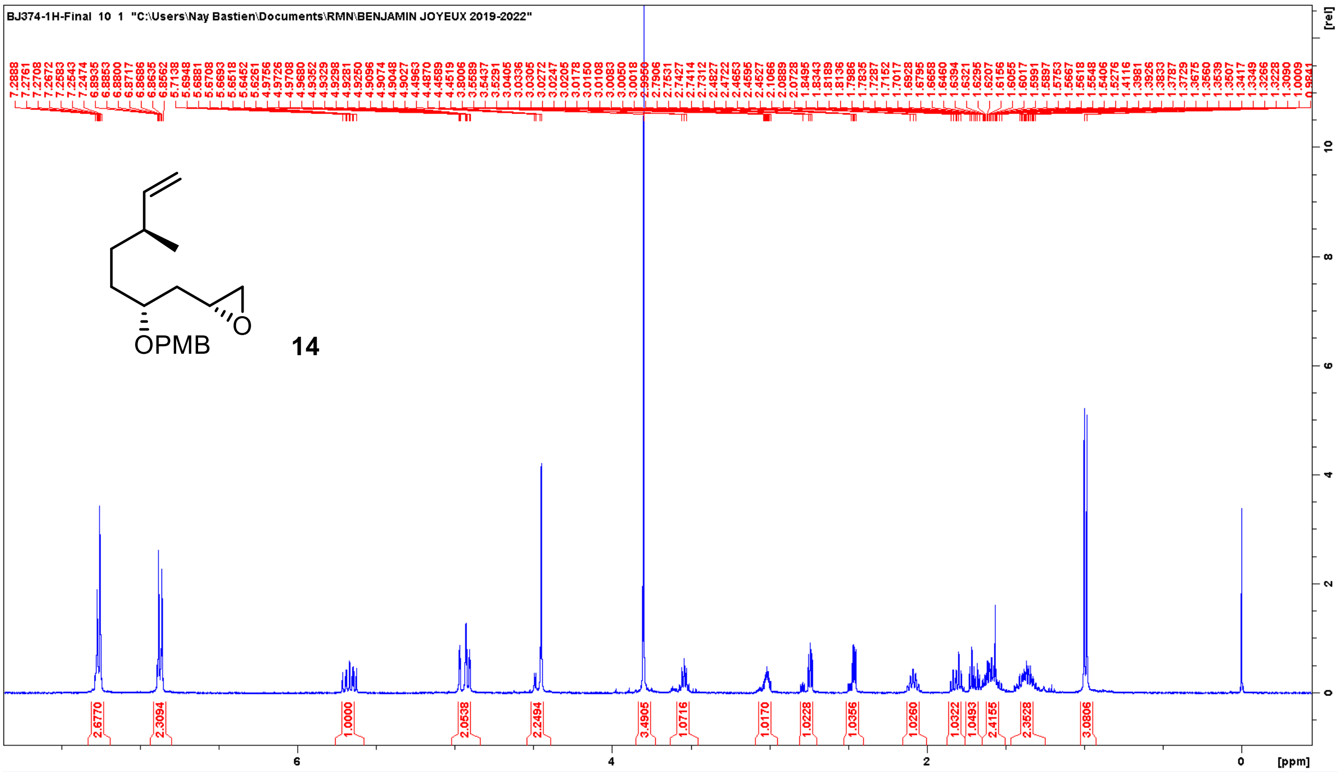

^{13}C NMR (100 MHz, $CDCl_3$)

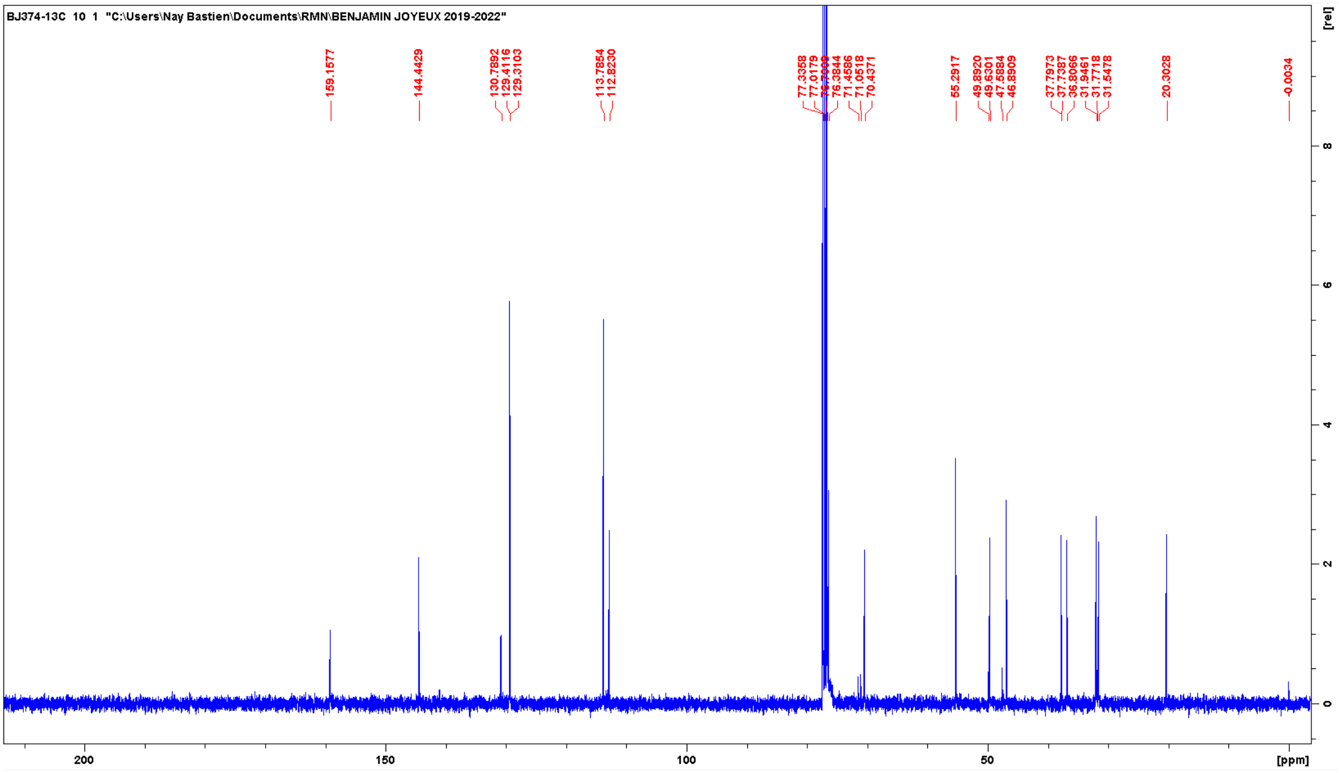

Intermediate S3

^{1}H NMR (400 MHz, $CDCl_3$)

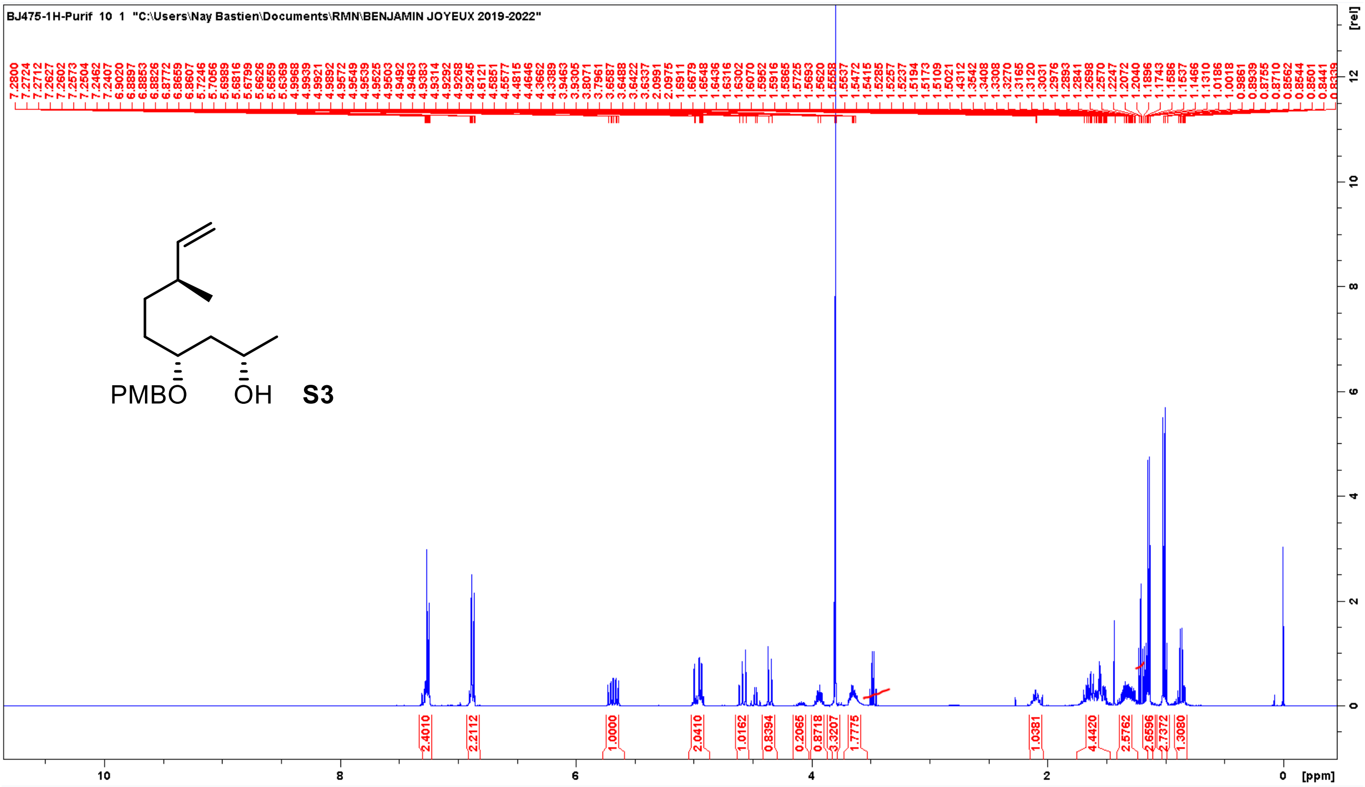

(4*R*,7*S*)-7-Methyl-4-(4-methoxybenzyloxy)nona-8-en-2-one (15)

^{1}H NMR (400 MHz, $CDCl_3$)

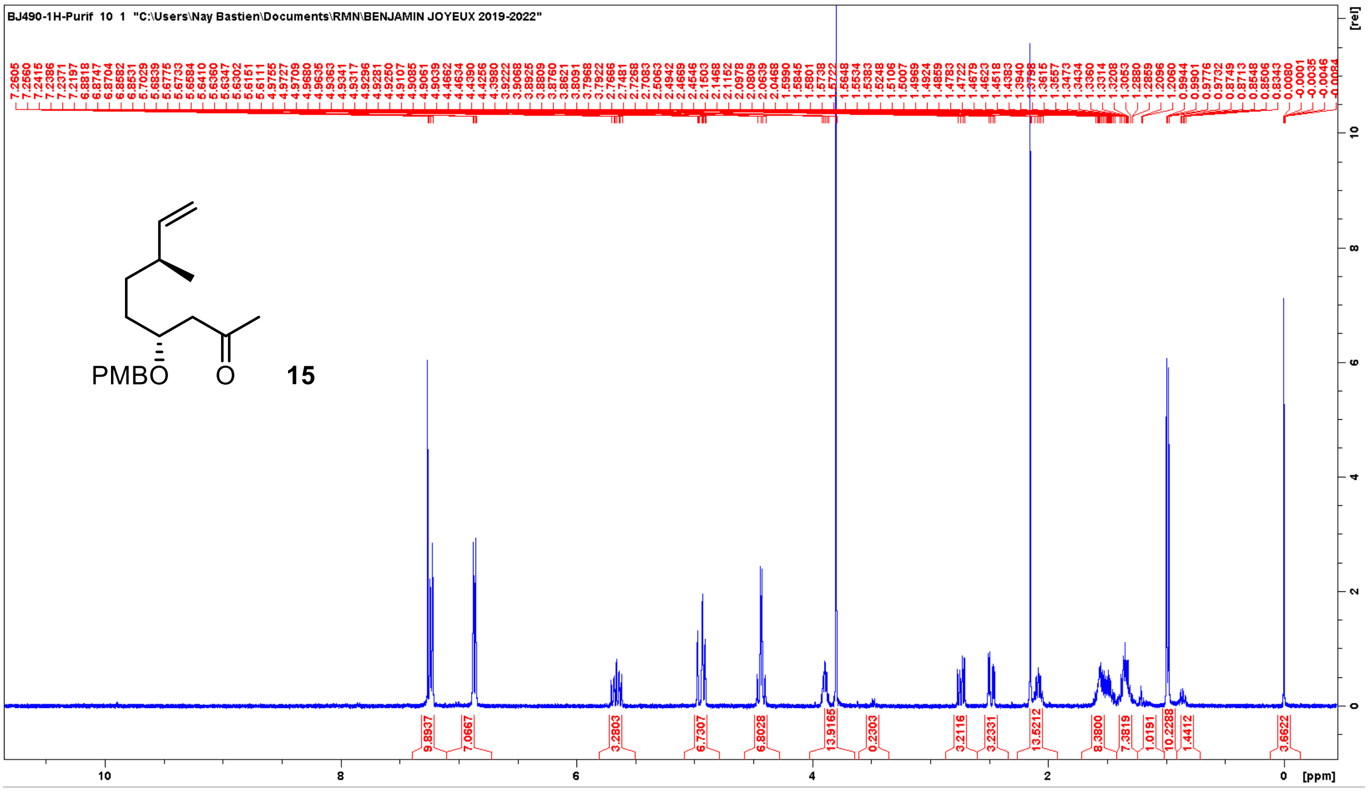

^{13}C NMR (100 MHz, $CDCl_3$)

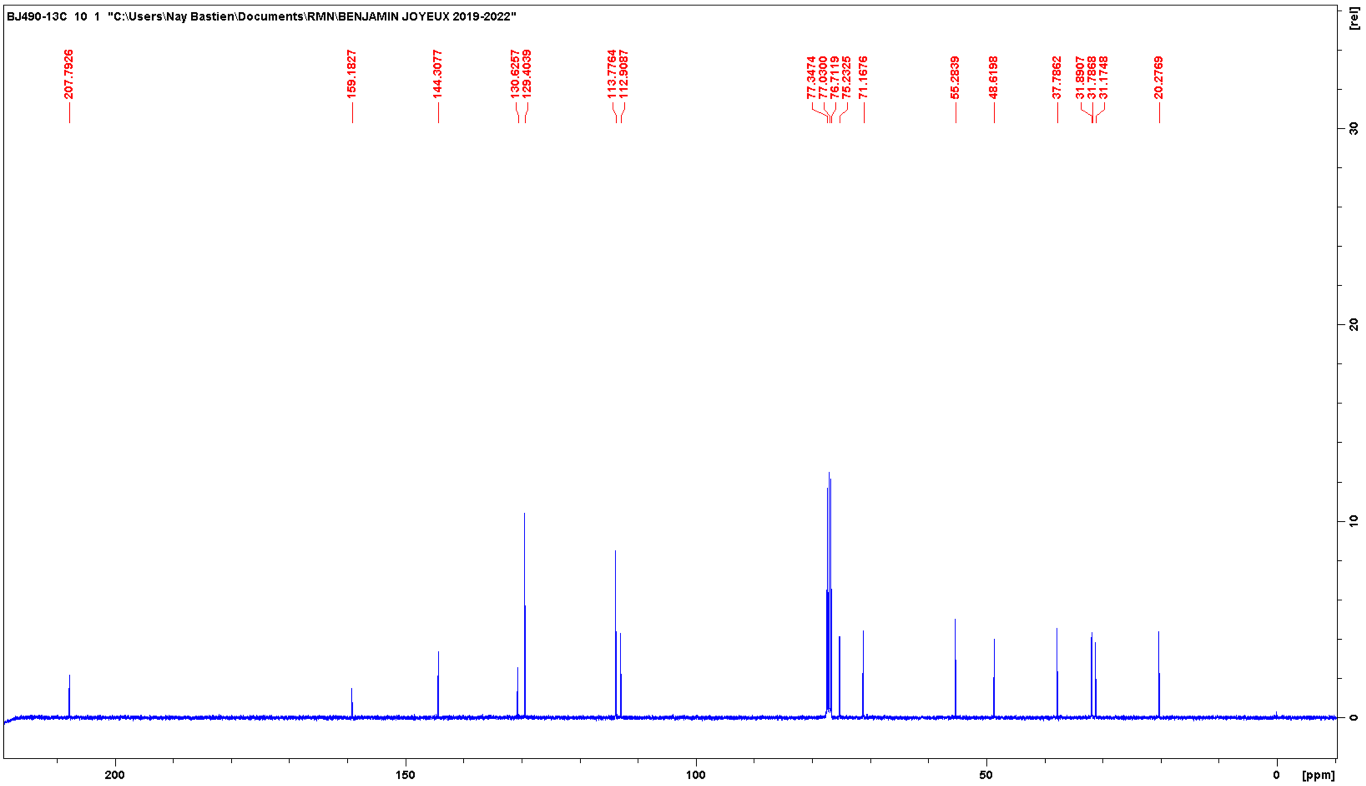

Ethyl (*R*)-2-oxothiazolidine-4-carboxylate (17)

^{1}H NMR (400 MHz, CDCl$_3$)

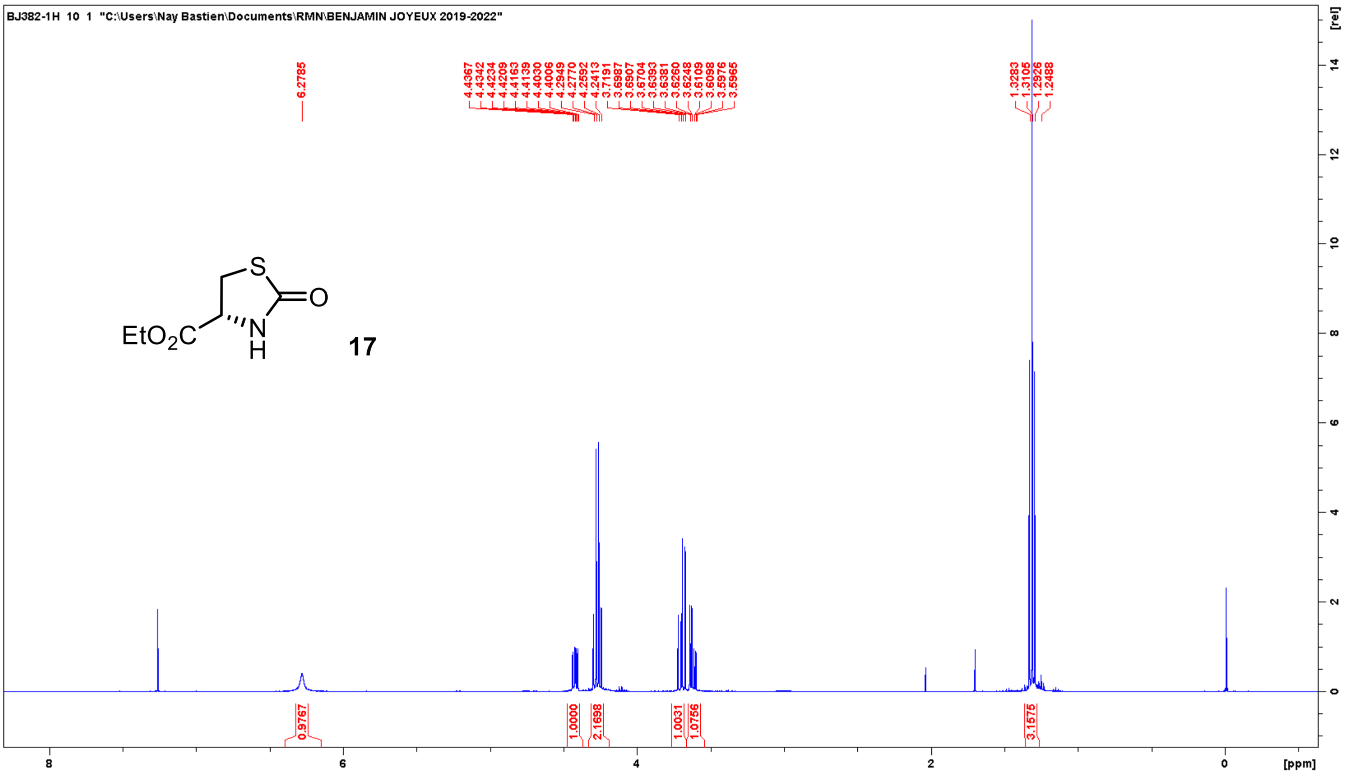

^{13}C NMR (100 MHz, CDCl$_3$)

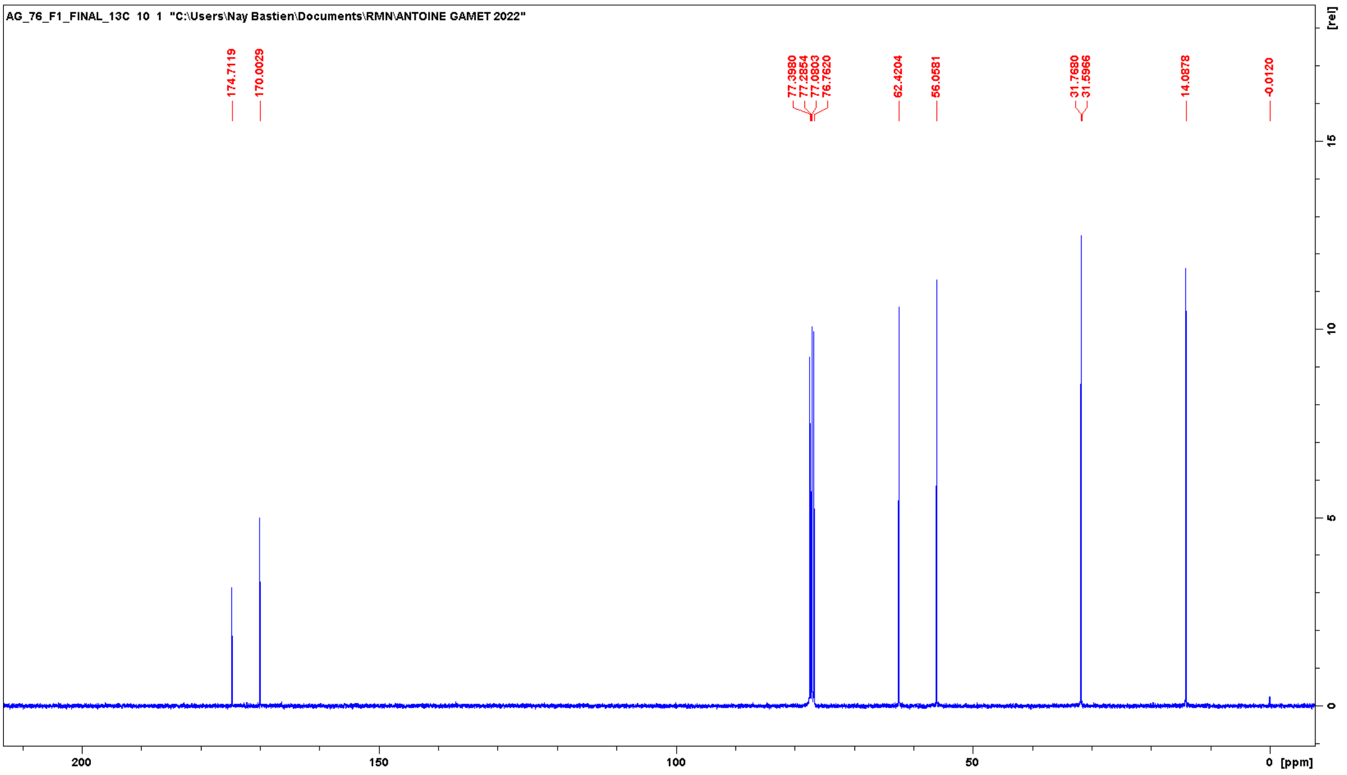

Ethyl (*R*)-*N*-(4-methoxybenzyl)-2-oxothiazolidine-4-carboxylate (18)

^{1}H NMR (400 MHz, $CDCl_3$)

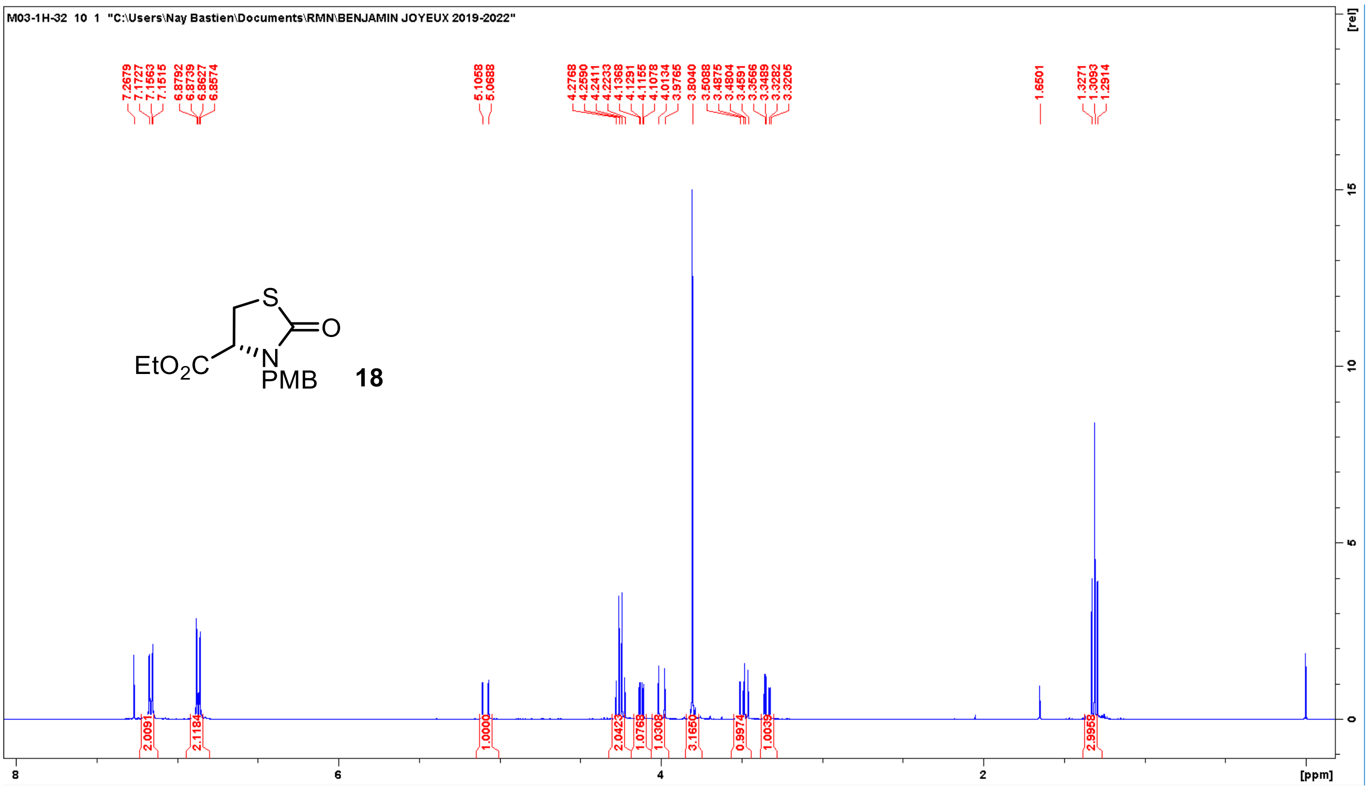

^{13}C NMR (100 MHz, $CDCl_3$)

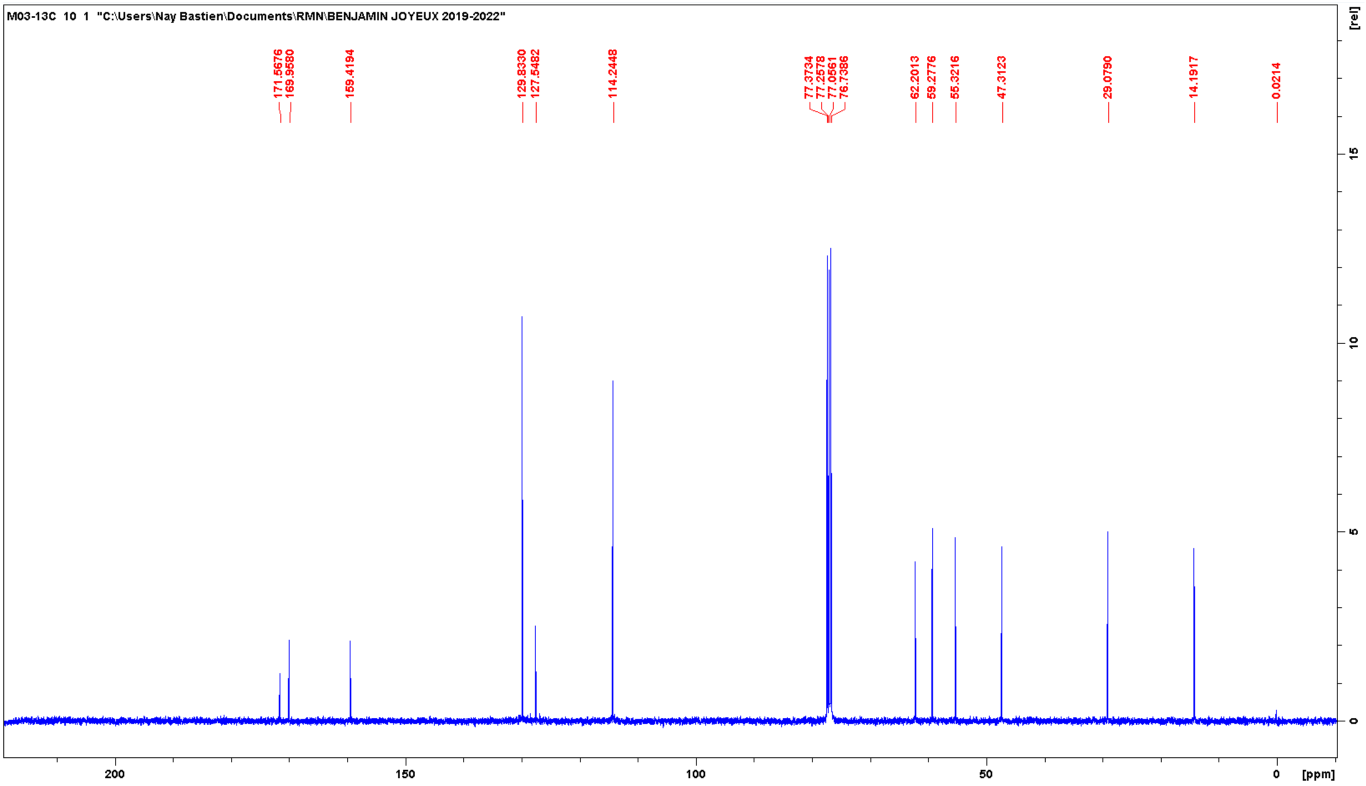

(*R*)-4-(Hydroxymethyl)-3-(4-methoxybenzyl)thiazolidin-2-one (19)

^{1}H NMR (400 MHz, $CDCl_3$)

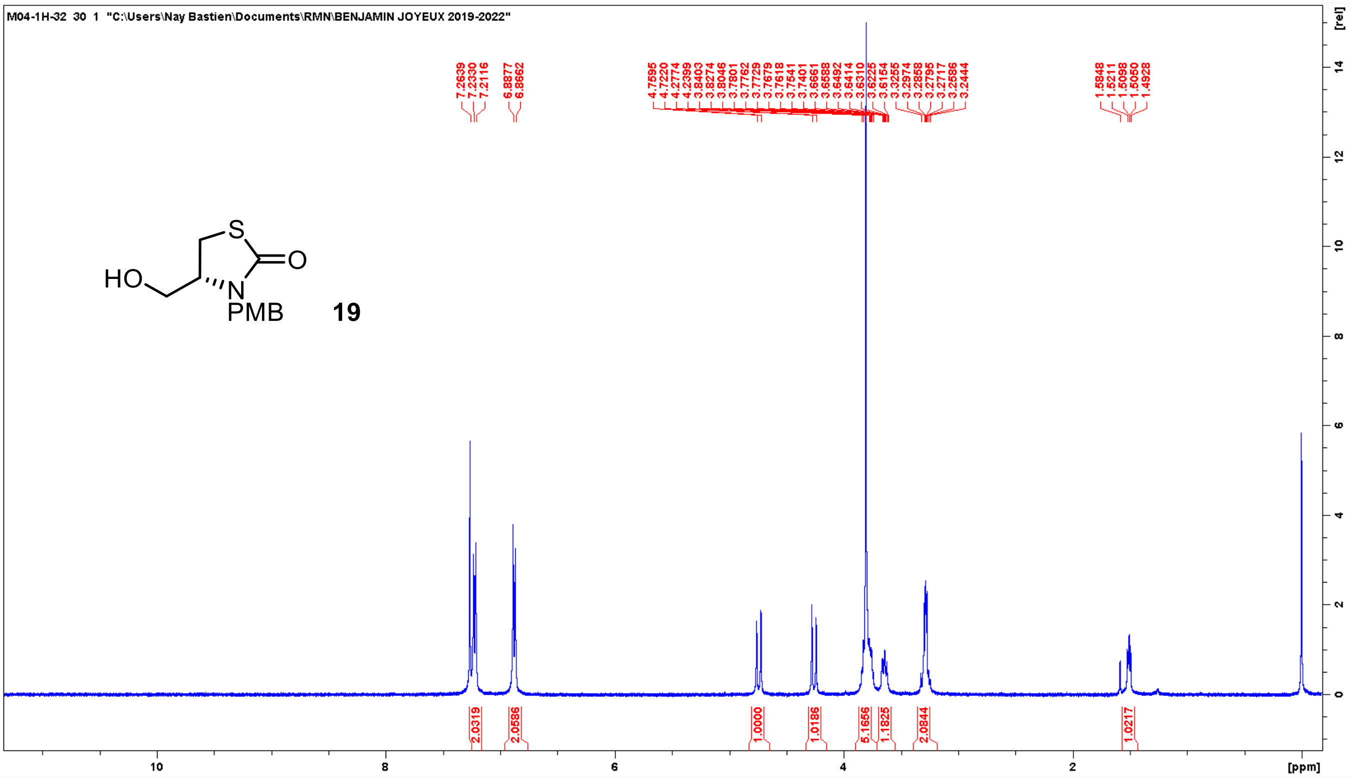

^{13}C NMR (100 MHz, $CDCl_3$)

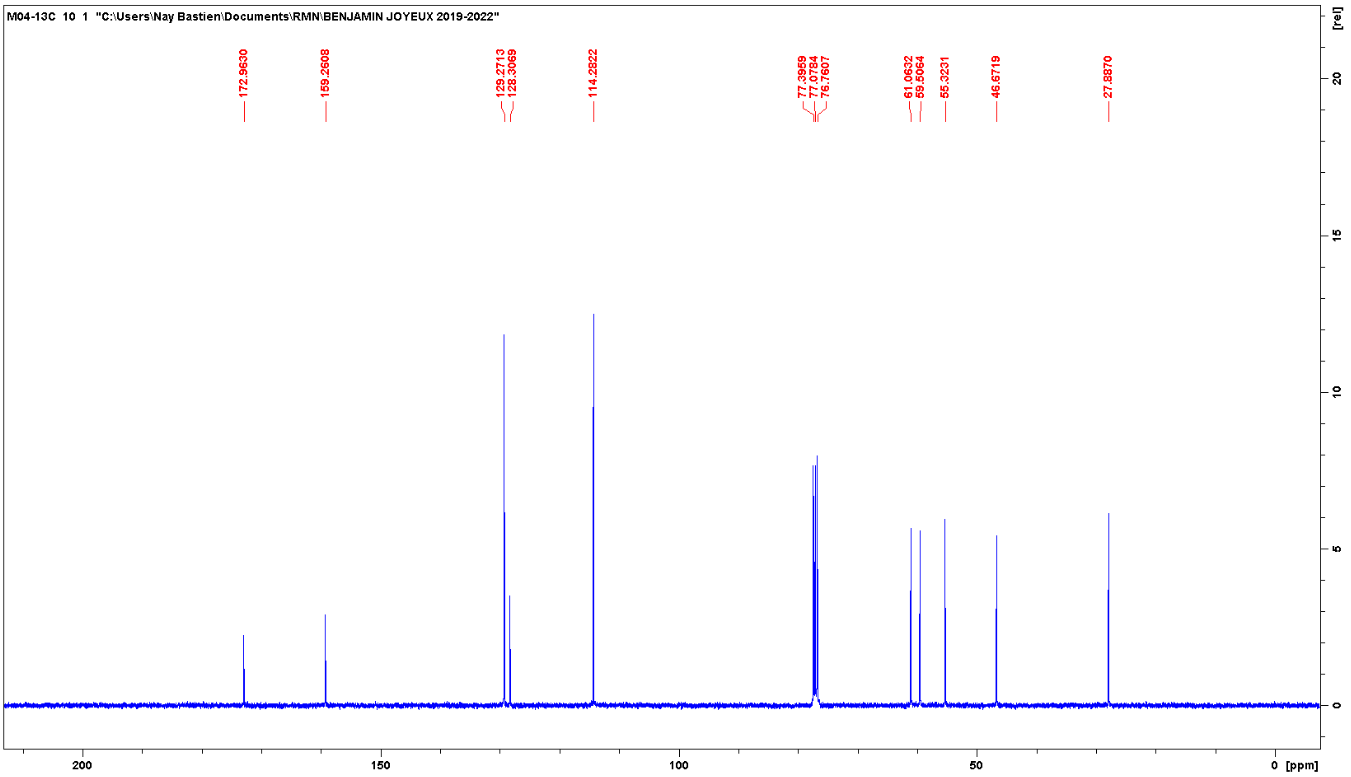

(*R*)-3-(4-Methoxybenzyl)-2-oxothiazolidine-4-carbaldehyde (8)

^{1}H NMR (400 MHz, $CDCl_3$)

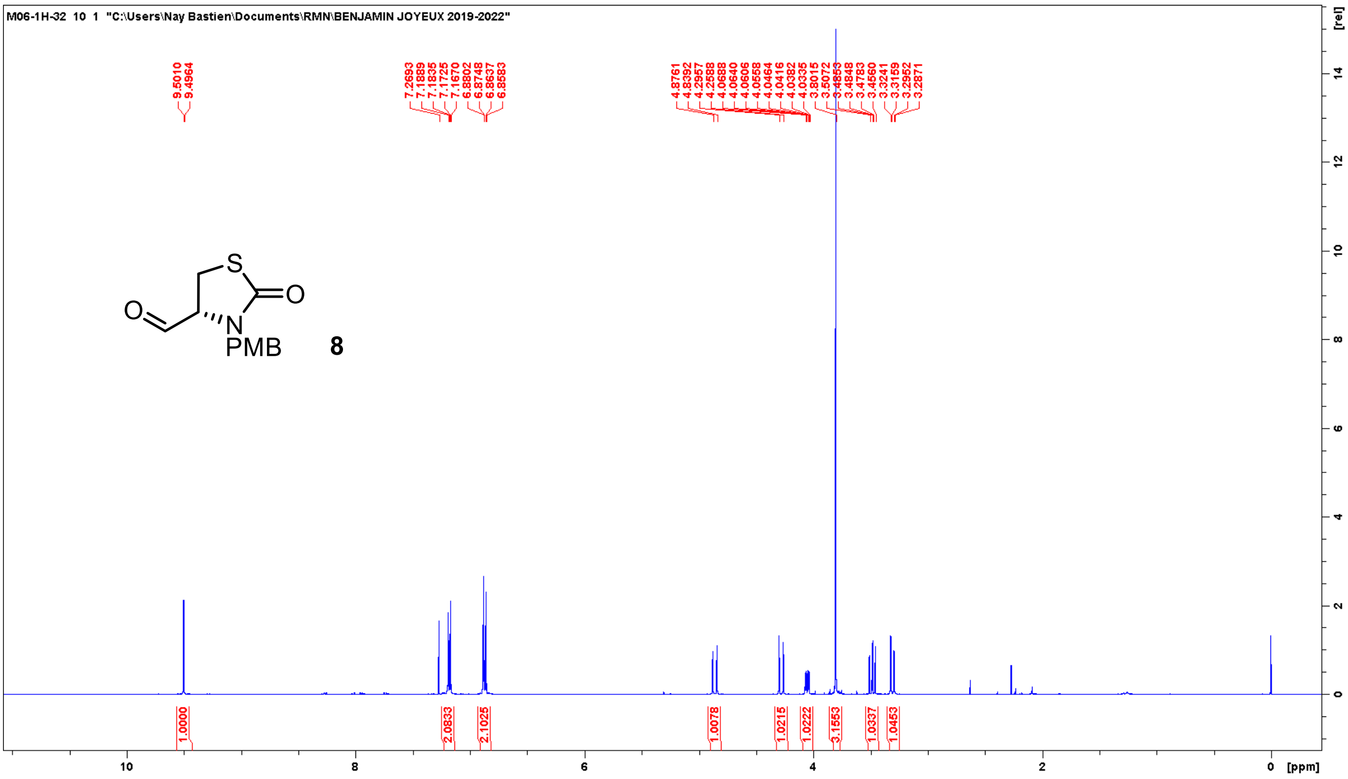

^{13}C NMR (100 MHz, $CDCl_3$)

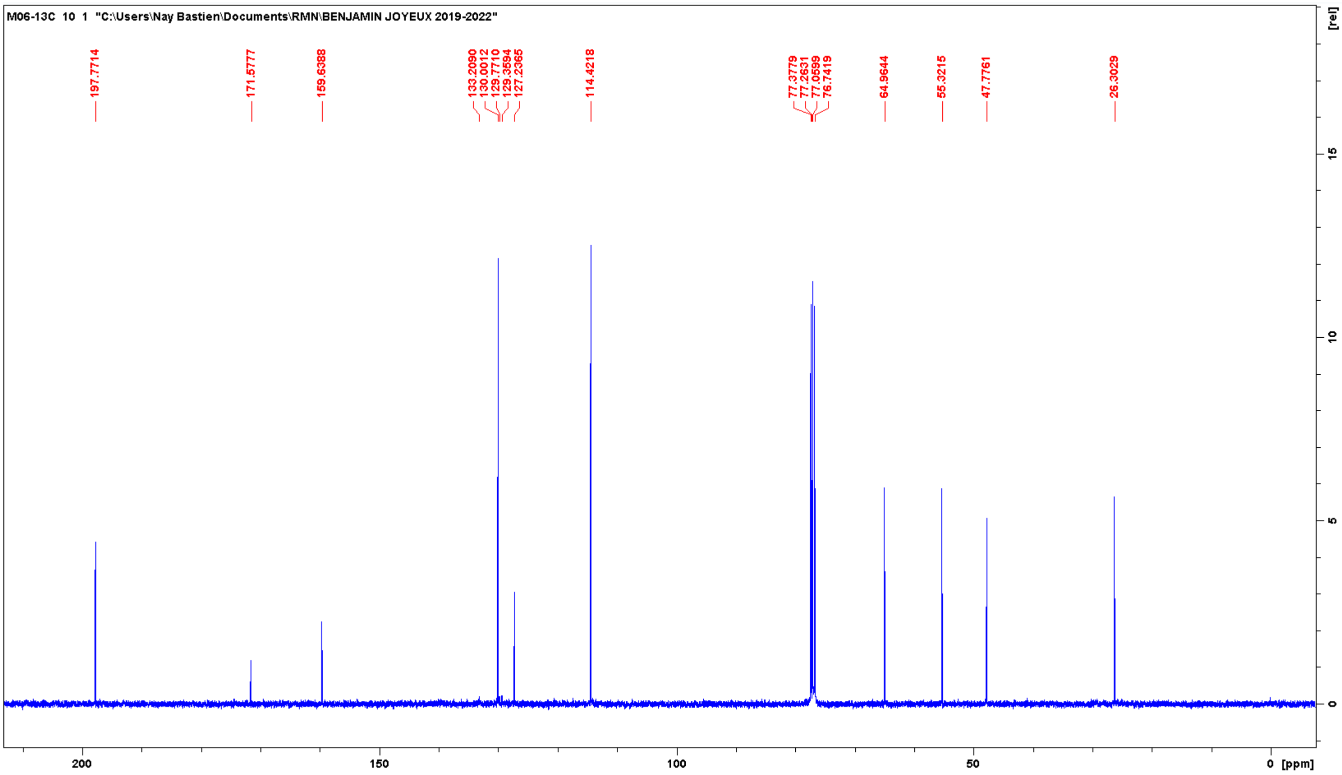

(*R*)-4-((1*R*,5*R*,8*S*)-1-Hydroxy-5-((4-methoxybenzyl)oxy)-8-methyl-3-oxodec-9-en-1-yl)-3-(4-methoxybenzyl)thiazolidin-2-one (21)

^{1}H NMR (400 MHz, $CDCl_3$)

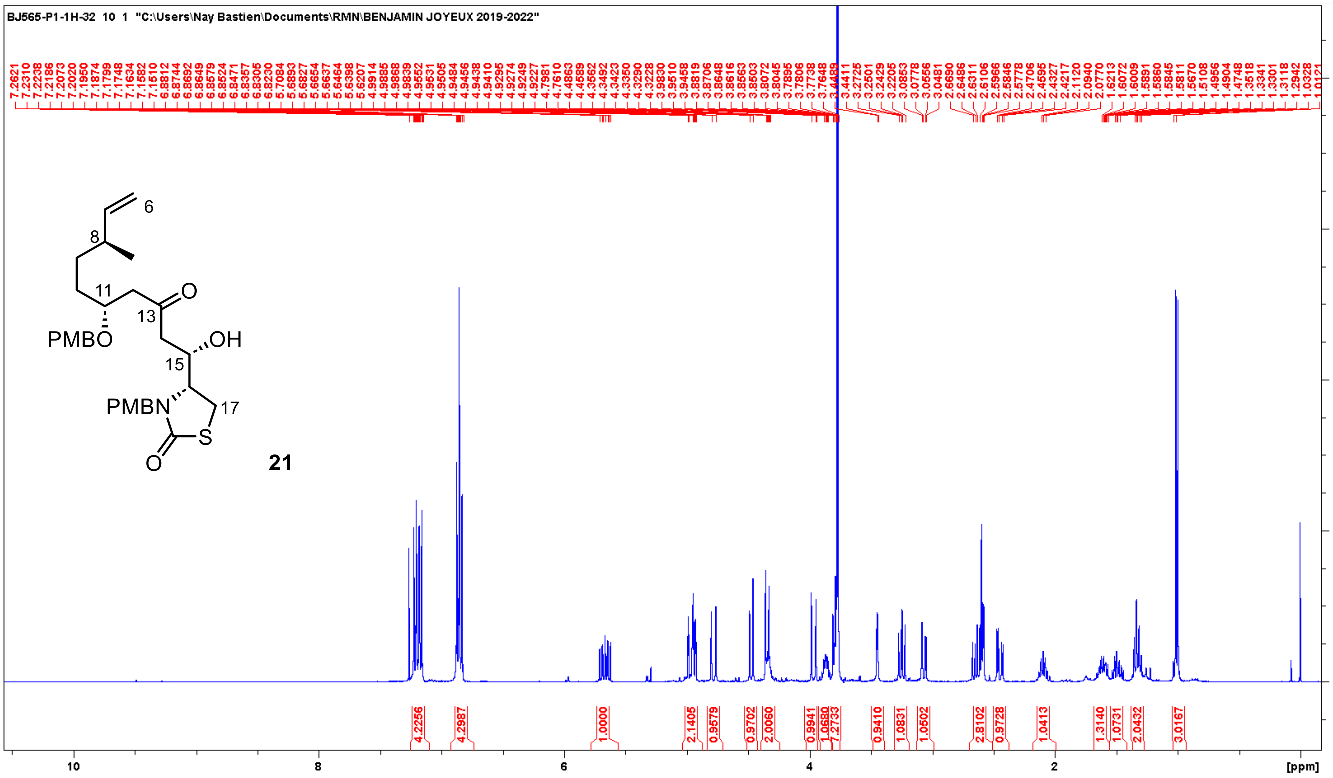

^{13}C NMR (100 MHz, $CDCl_3$)

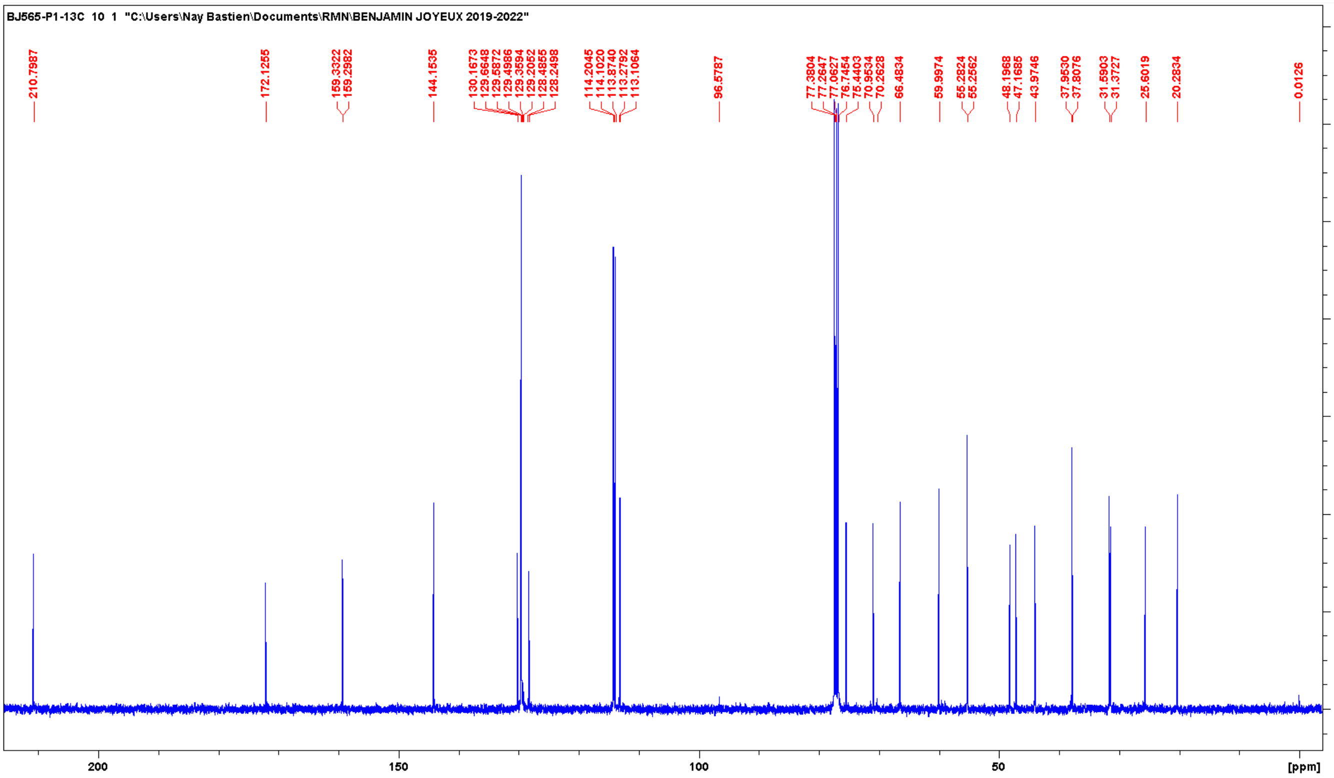

(1*R*,3*R*,5*R*,8*S*)-1-Hydroxy-1-((*R*)-3-(4-methoxybenzyl)-2-oxothiazolidin-4-yl)-5-((4-methoxybenzyl)oxy)-8-methyldec-9-en-3-yl 4-nitrobenzoate (23)

^{1}H NMR (400 MHz, $CDCl_3$)

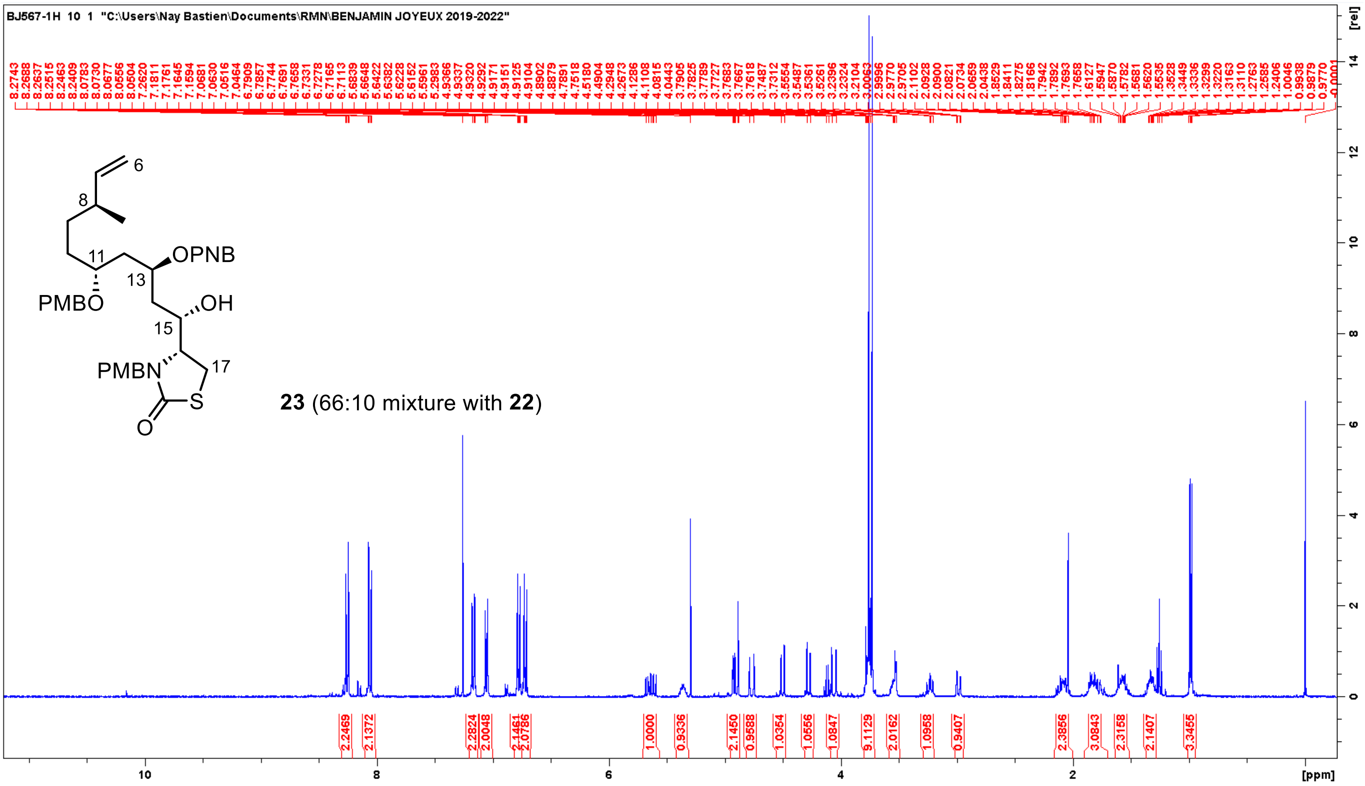

^{13}C NMR (100 MHz, $CDCl_3$)

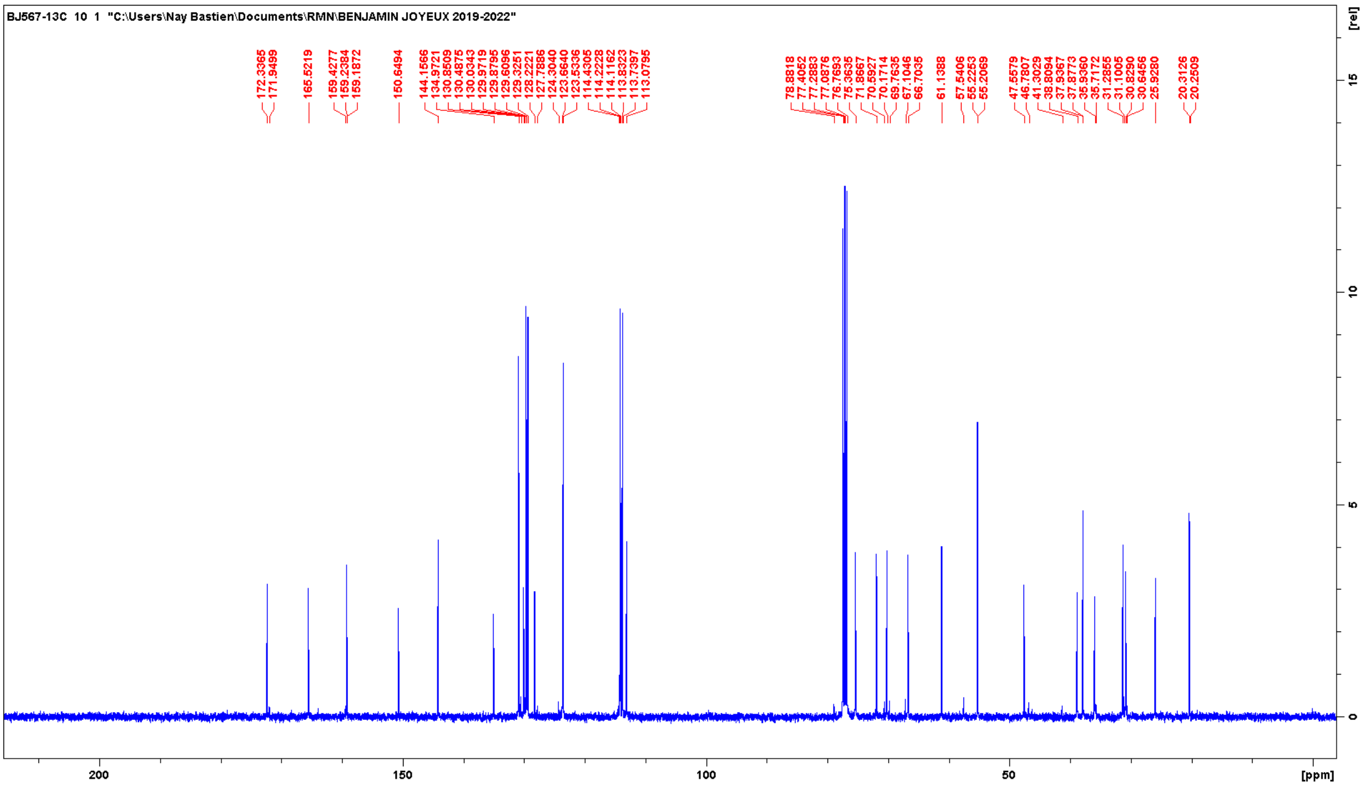

Data for minor isomer 22 (ca. 3:1 mixture with 23)

^{1}H NMR (400 MHz, CDCl$_3$)

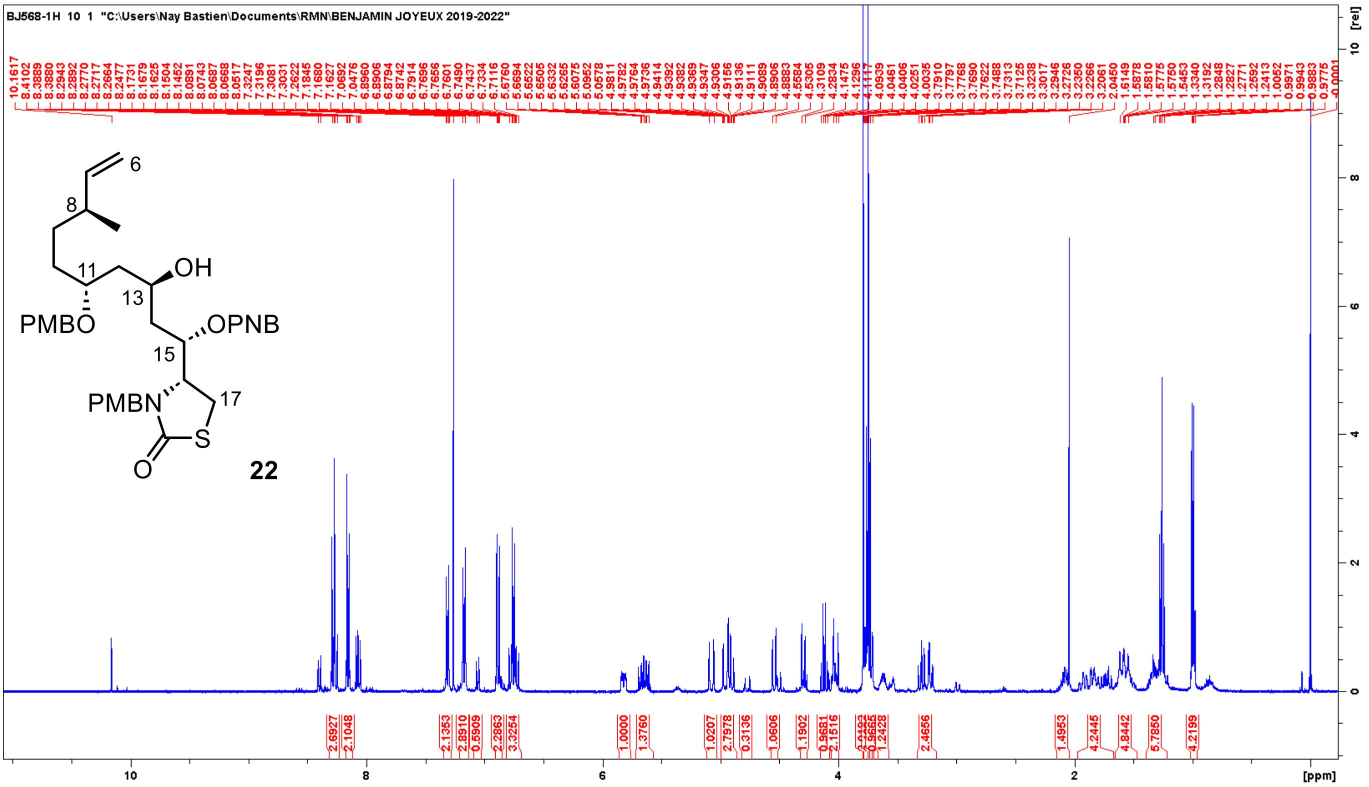

^{13}C NMR (100 MHz, CDCl$_3$)

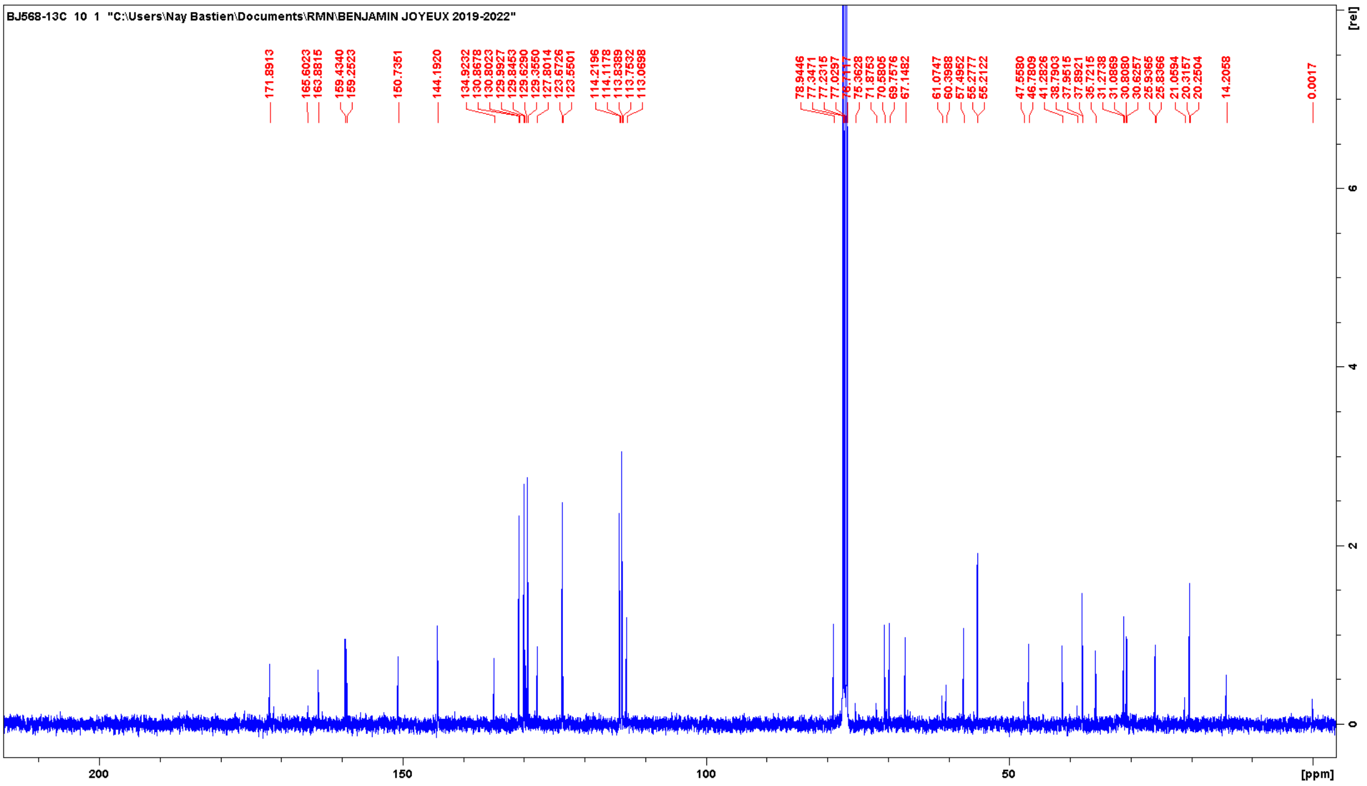

(*R*)-4-((1*S*,3*R*,5*R*,8*S*)-1,3-Dihydroxy-5-((4-methoxy-benzyl)oxy)-8-methyldec-9-en-1-yl)-3-(4-methoxybenzyl)thiazolidin-2-one (24)

^{1}H NMR (400 MHz, $CDCl_3$)

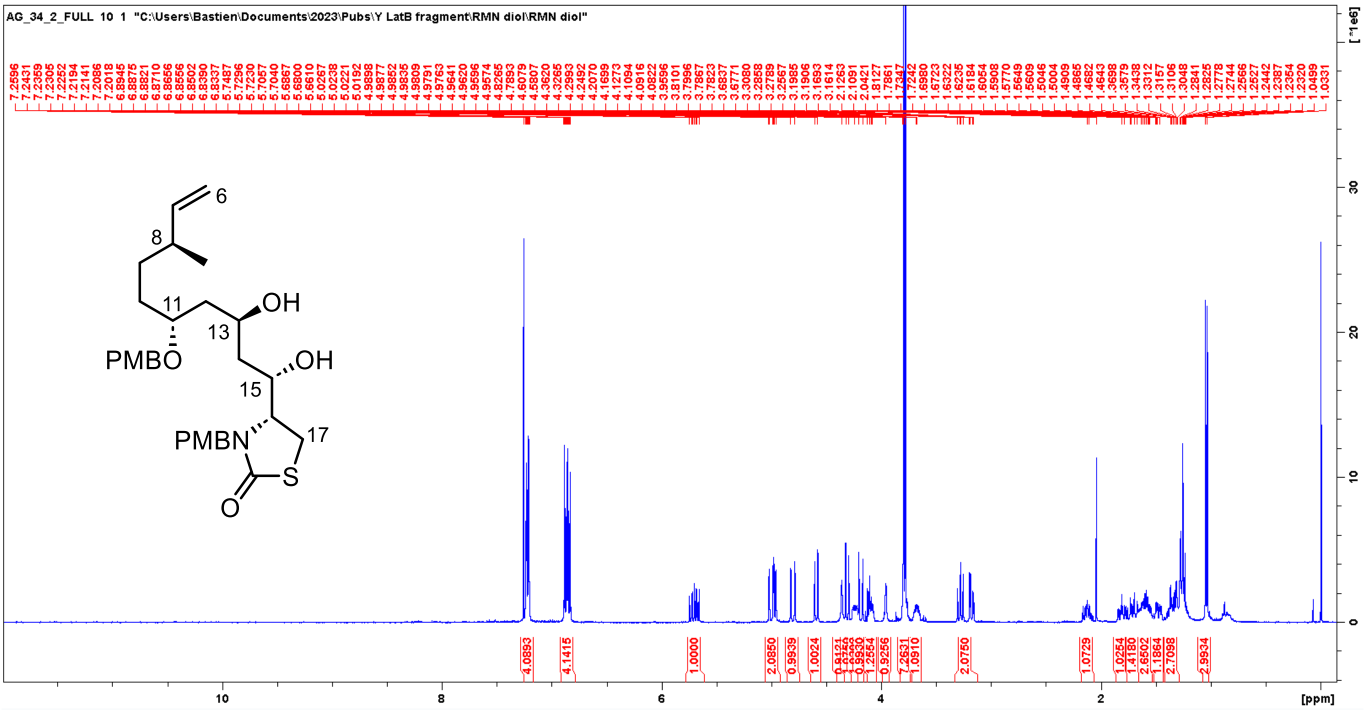

^{13}C NMR (100 MHz, $CDCl_3$)

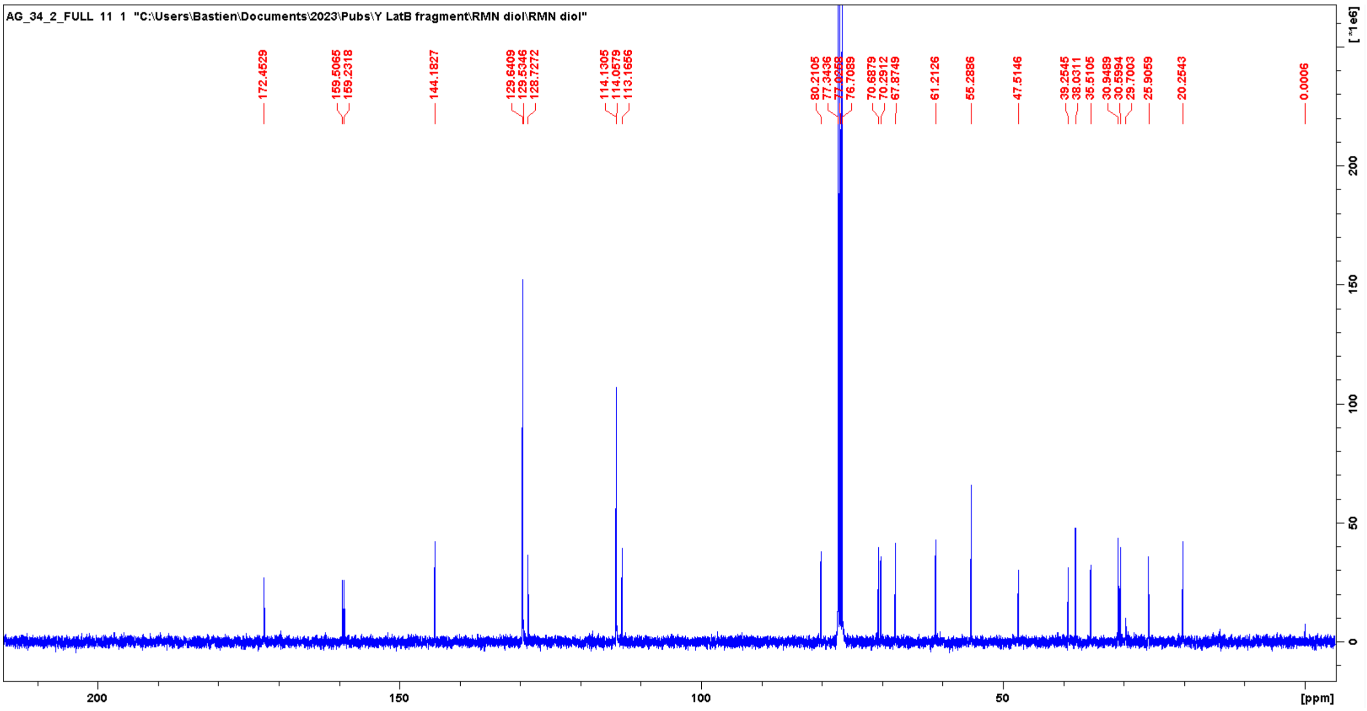

(*R*)-4-((1*S*,3*R*,5*R*,8*S*)-1,3-((*R*)-(4-Methoxyphenyl)methylenedioxy)-5-((4-methoxy-benzyl)oxy)-8-methyldec-9-en-1-yl)-3-(4-methoxybenzyl)thiazolidin-2-one (25)

^{1}H NMR (400 MHz, $CDCl_3$)

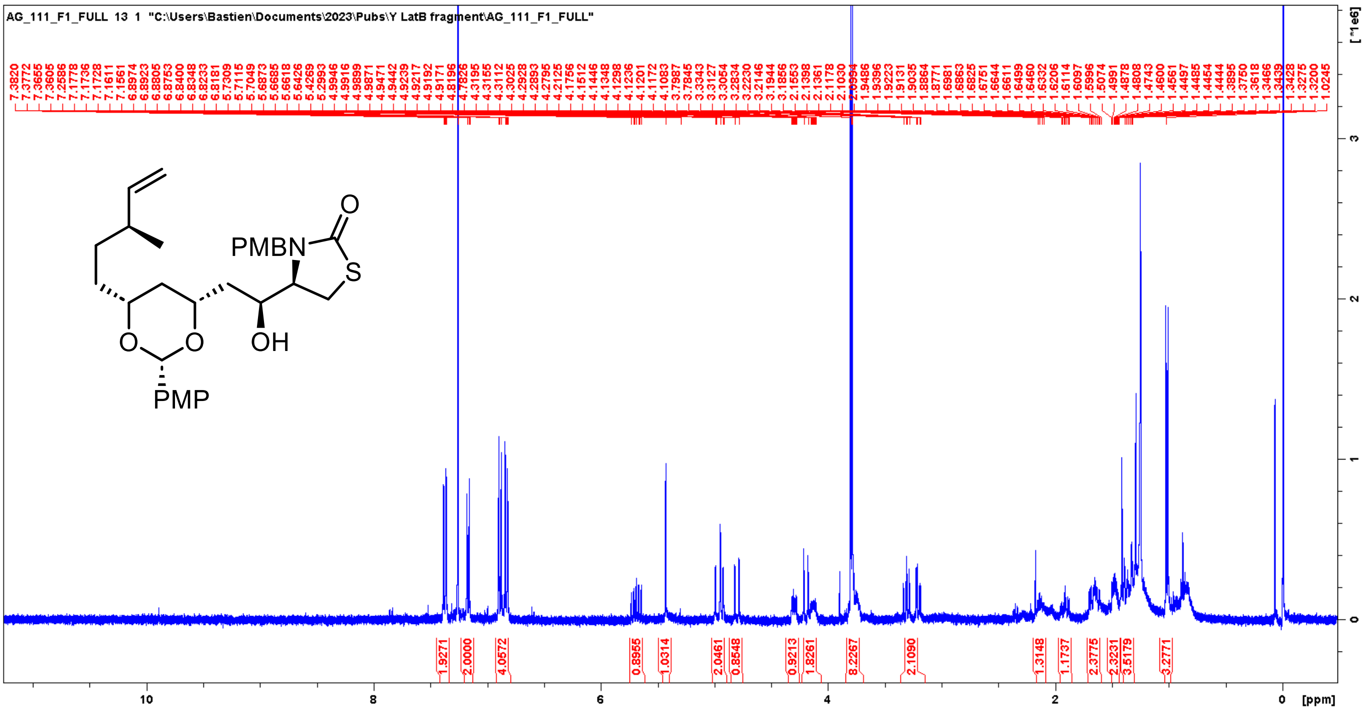

^{13}C NMR (100 MHz, $CDCl_3$)

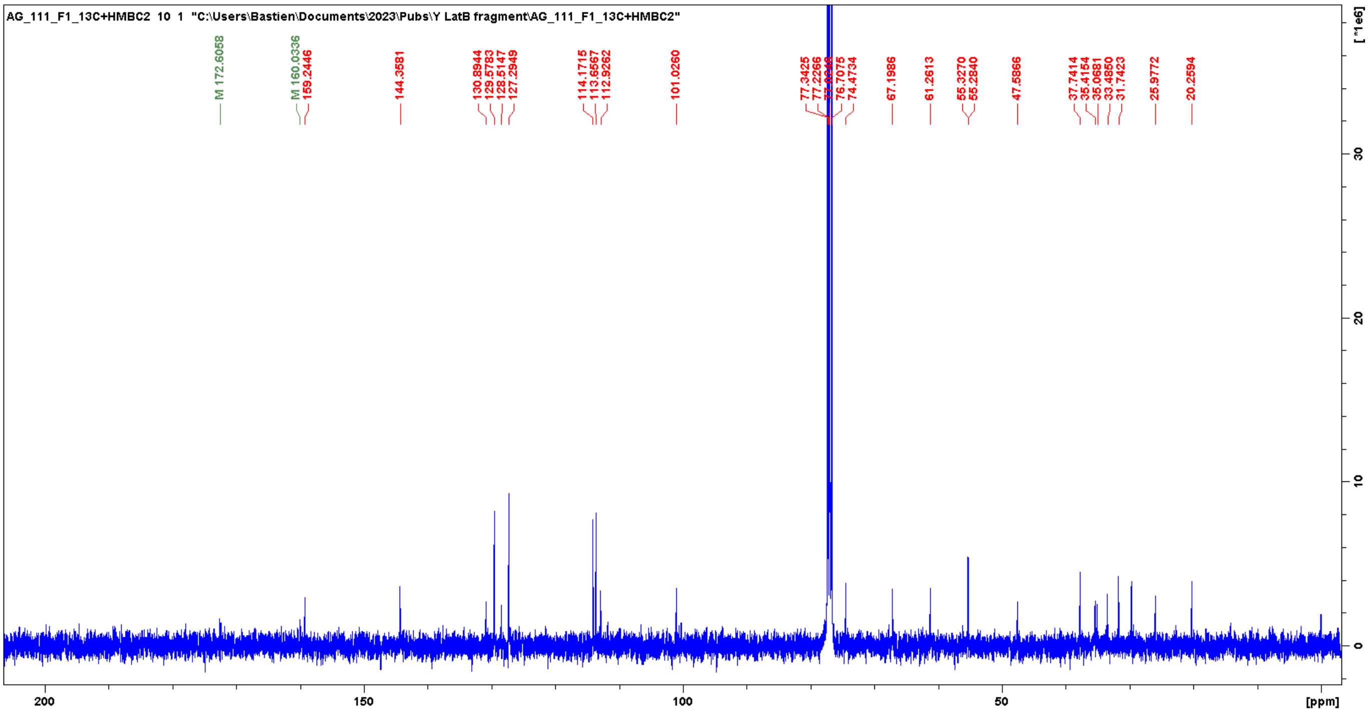

COSY NMR ($CDCl_3$)

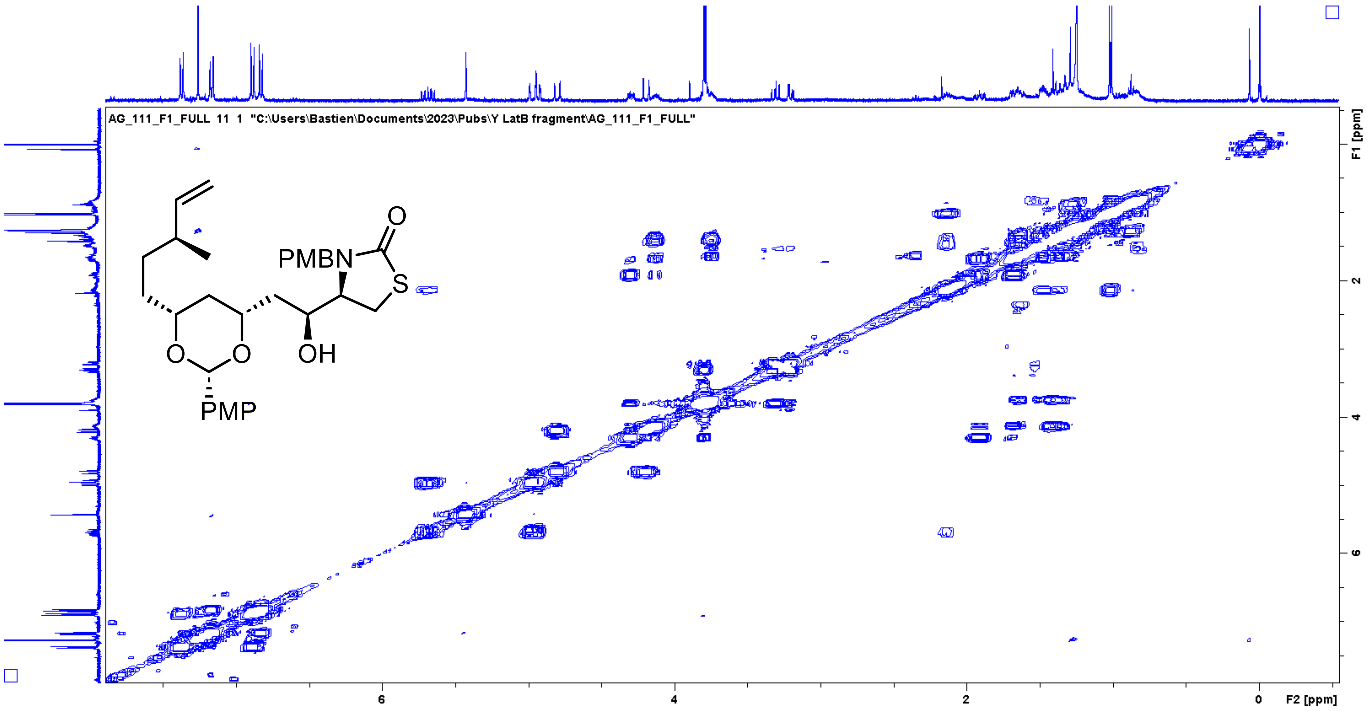

NOESY NMR ($CDCl_3$)

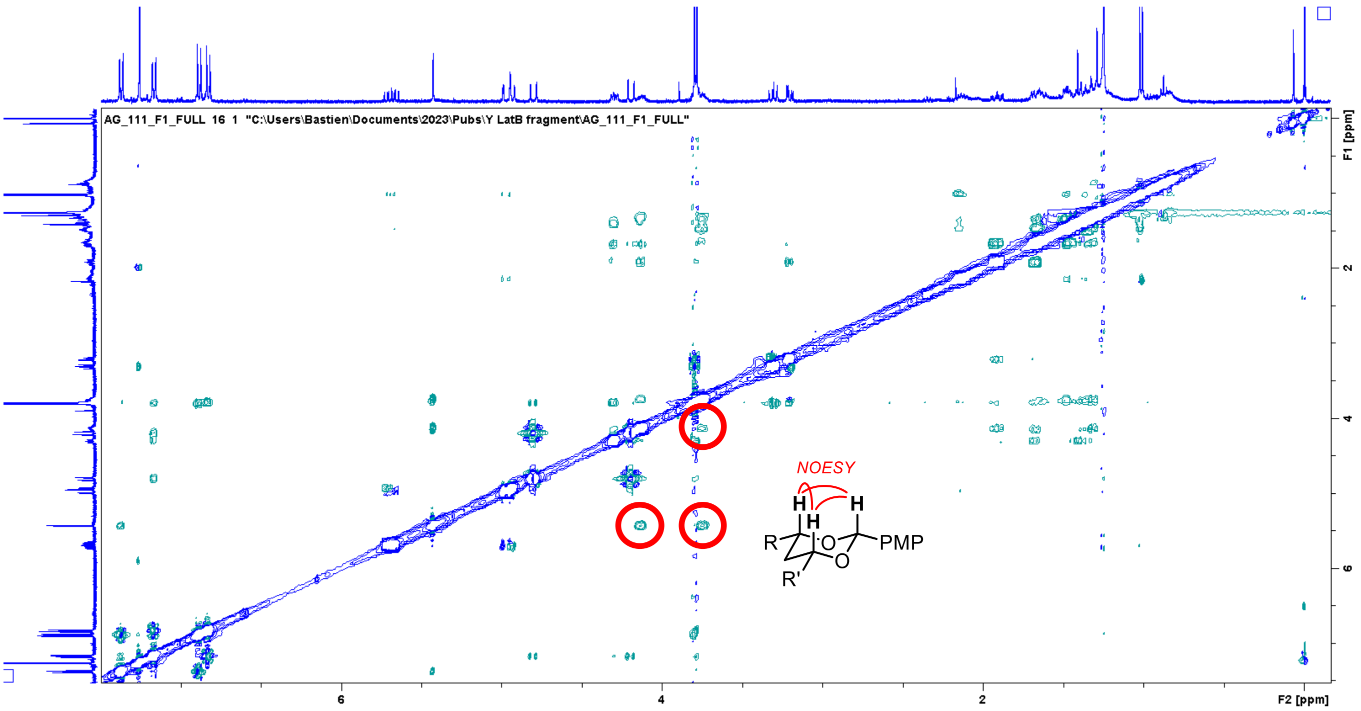

Supplement: File 1 — Experimental procedures, compound characterizations and spectra. [file Beilstein_J_Org_Chem-19-428-s001.pdf]
